# Supplementary material for: Efficient and scalable generation of primordial germ cells in 2D culture using basement membrane extract overlay
Source: Cell Rep Methods. 2023 May 23;3(6):100488. doi: 10.1016/j.crmeth.2023.100488 (PMC10326346; doi:10.1016/j.crmeth.2023.100488)
Supplement: Document S1. Figures S1–S4 [file mmc1.pdf]

Cell Reports Methods, Volume *3*

Supplemental information

Efficient and scalable generation of primordial germ cells in 2D culture using basement membrane extract overlay

Arend W. Overeem, Yolanda W. Chang, Ioannis Moustakas, Celine M. Roelse, Sanne Hillenius, Talia Van Der Helm, Valérie F. Van Der Schrier, Manuel A.F.V. Gonçalves, Hailiang Mei, Christian Freund, and Susana M. Chuva de Sousa Lopes

SUPPLEMENTAL INFORMATION

SUPPLEMENTAL FIGURES AND FIGURE LEGENDS

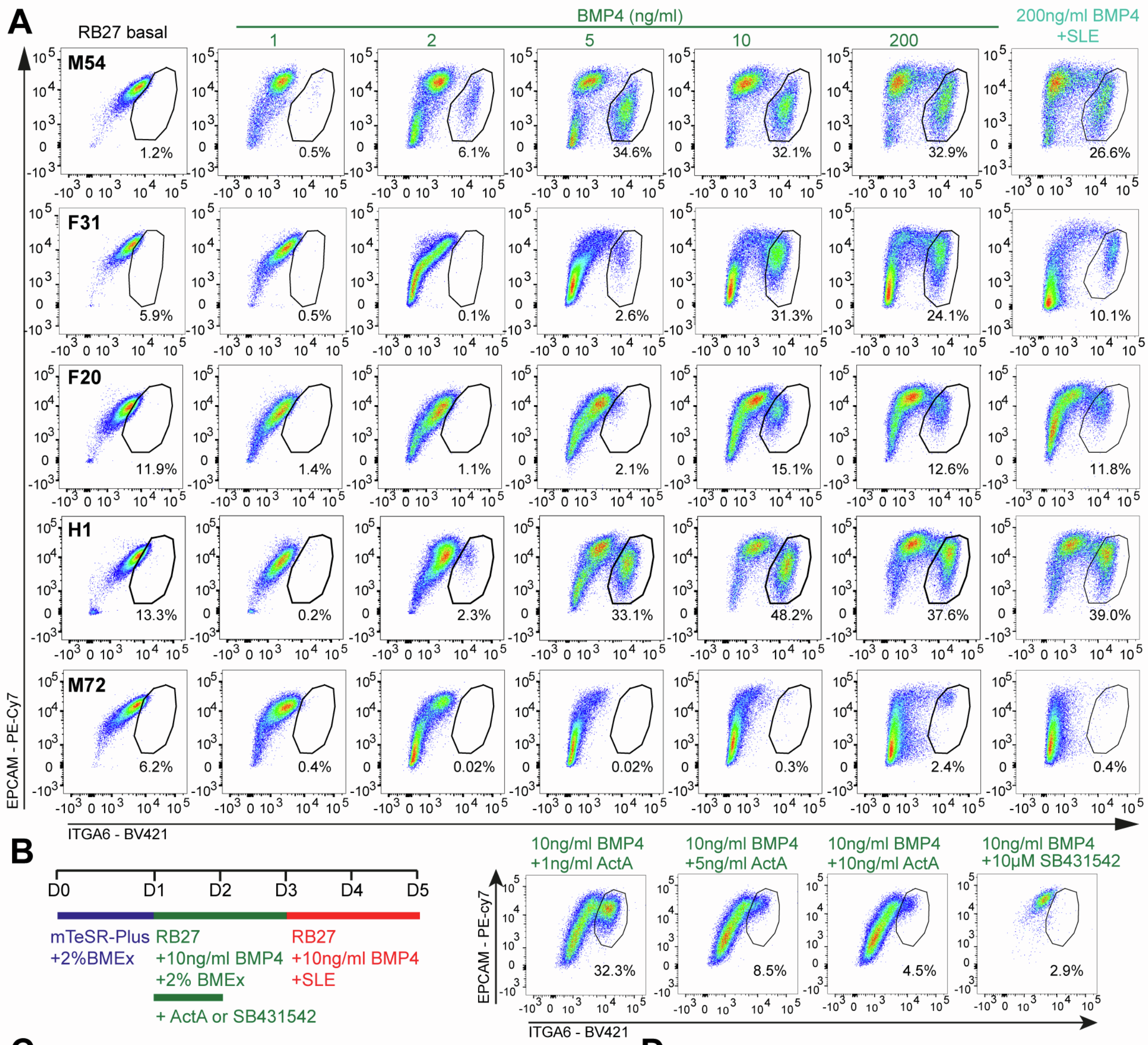

C

	Label	Lot number	BCA assay (mg/ml)	Lowry assay from product sheet (mg/ml)
Geltrex	Lot A	963718	7.0	13.0
	Lot B	963726	5.9	16.0
	Lot C	2327546	6.4	13.2
Cultrex	Lot A	1659223	8.4	9.6
	Lot B	1677279	7.2	9.8
Matrigel	Lot A	1341001	8.8	6.5
	Lot B	1235001	9.1	9.0

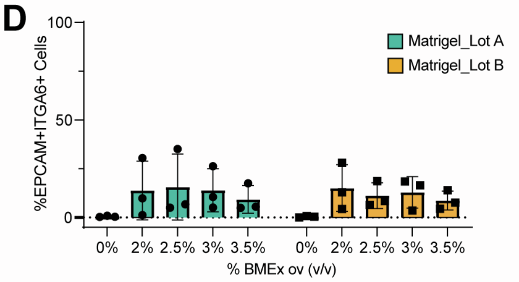

Figure S1. hPGCLC differentiation using BMEx overlay method across multiple cell lines. Related to Figure 2.

(A) FACS plots depicting the percentage of EPCAM+ITGA6+ cells at D5 in different lines to test different BMP4 concentrations.

(B) Experimental scheme depicting different conditions tested (left) and the associated FACS plots (right) depicting the percentage of EPCAM+ITGA6+ cells at D5 in line F99.

(C) Protein concentrations and lot numbers of all the BMEx products used in this study.

(D) Bar graph showing mean percentages (mean ± standard deviation, SD) of EPCAM+ITGA6+ cells at D5 generated using different percentages of hESC-qualified Matrigel overlay comparing to no overlay control (0%), analyzed by FACS in line M54.

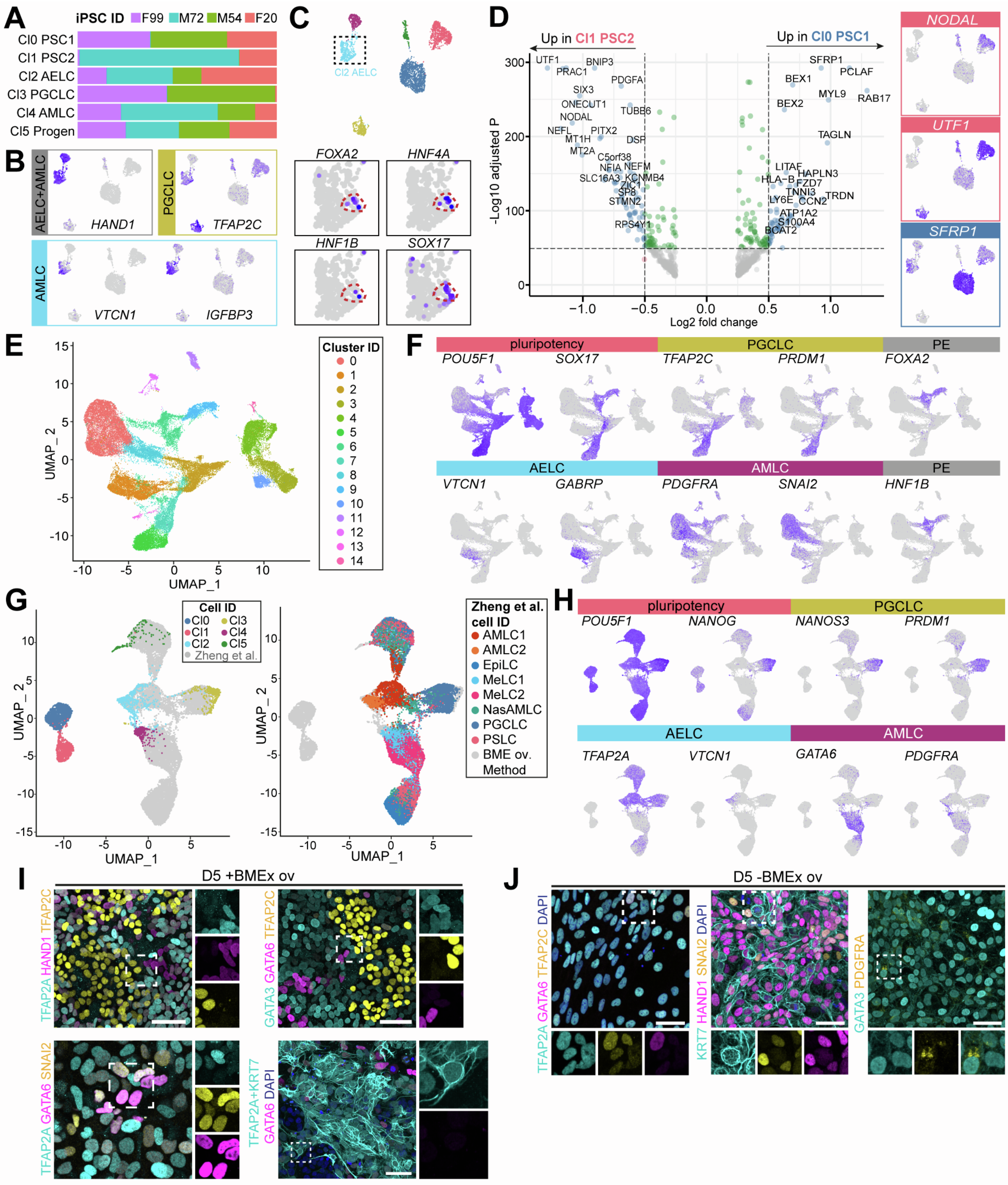

Figure S2. RNAseq comparing BMEx overlay method with EB method and µPASE method, and verification of markers by IF. Related to Figure 3.

(A) Bar graph depicting the contribution per cluster from each cell line.

(B) Expression of additional signature genes of cell types of interest on the UMAP plot from Figure 3A.

(C) Expression of signature genes of endoderm on the UMAP plot from Figure 3A (close up).

(D) Volcano plot showing DEGs between the two D0 hPSCs clusters (Cl0 and Cl1) (left panel) and expression of selected DEGs on the UMAP plot from Figure 3A (right panels).

(E) UMAP showing integrated single-cell transcriptomics data from EB-differentiation method (UCLA2 from Chen et al., 2019) and BMEx overlay method.

(F) Expression of signature genes of cell types of interest on the UMAP plot from Figure 3D.

(G) UMAP showing integrated single-cell transcriptomics data from amniotic sac embryoid system (μPASE) (from Zheng et al., 2022) and BMEx overlay method.
(H) Expression of signature genes of cell types of interest on the UMAP plots from Figure S3G.
(I) Immunofluorescence for TFAP2A, HAND1 and TFAP2C; GATA3, GATA6 and TFAP2C; TFAP2A, GATA6 and SNAI2; and TFAP2A+KRT7 and GATA6 at D5 with BMEx overlay. The dashed box is magnified showing separated channels. Scalebars: 50μm.
(J) Immunofluorescence for TFAP2A, GATA6 and TFAP2C; KRT7, HAND1 and SNAI2; GATA3 and PDGFRA at D5 without BMEx overlay. The dashed box is magnified showing separated channels. Scalebars: 50μm.

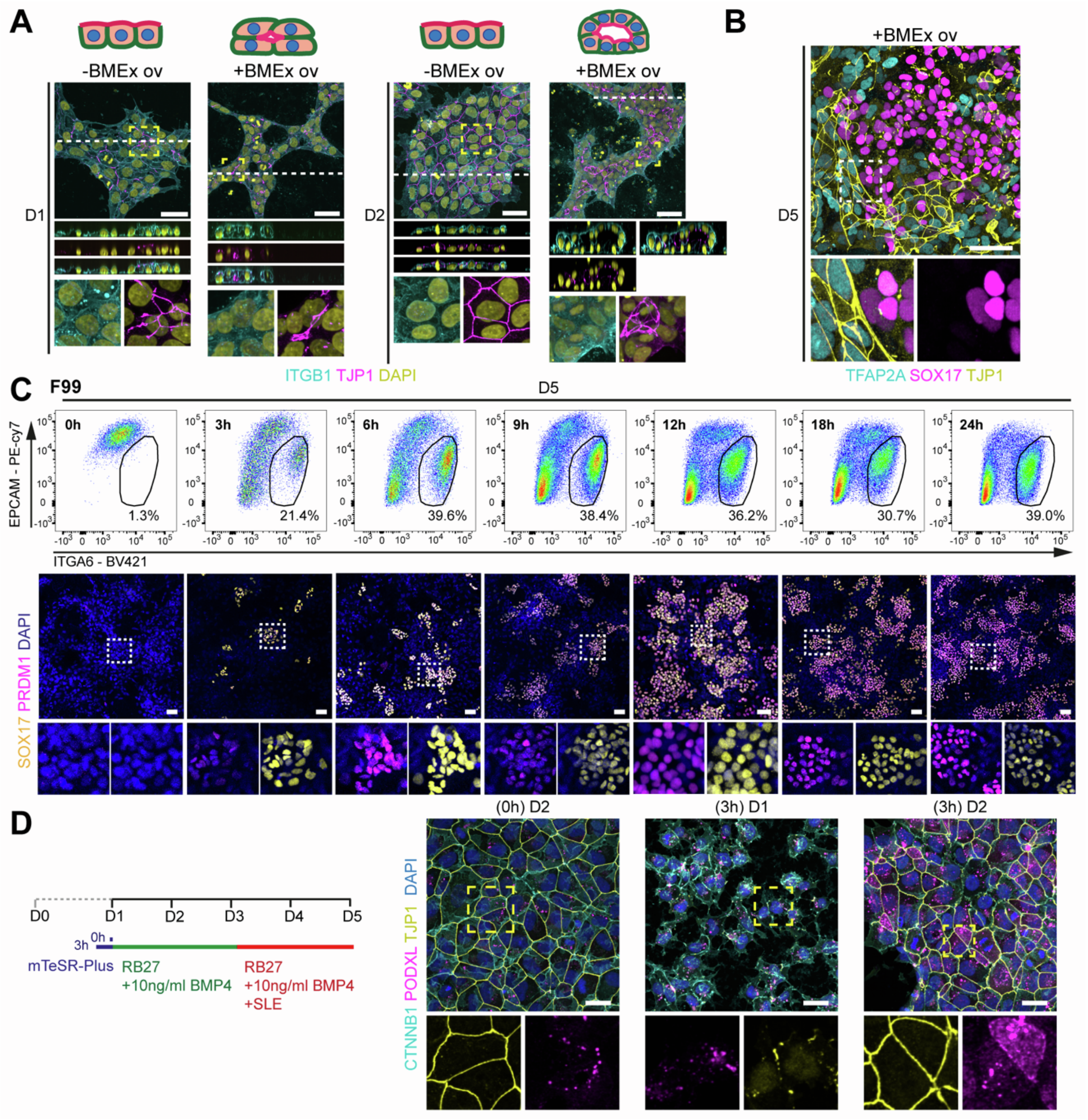

Figure S3. Lumenogenesis during hPGCLC differentiation with BMEx overlay. Related to Figure 5.

(A) Immunofluorescence for ITGB1 and TJP1 at D1 and D2 with or without BMEx overlay. White dashed line shows the level of the digital cross section (middle panels) and yellow dashed box is magnified (bottom) showing separated channels. Scale bars: 30μm.

(B) Immunofluorescence for TFAP2A, SOX17 and TJP1 at D5 with BMEx overlay. Dashed box is magnified (bottom) showing separated channels. Scale bars: 50μm.

(C) FACS plots depicting the percentage of double EPCAM+ITGA6+ cells at D5 in line F99 to test different priming periods (top) and associated immunofluorescence for SOX17 and PRDM1. Dashed box is magnified (bottom) showing separated channels. Scale bars: 50μm.

(D) No BMEx overlay control accompanying Figure 5G. Experimental scheme depicting the conditions tested in the absence of BMEx overlay (left) and immunofluorescence for CTNNB1, PODXL, TJP1 at D1 and D2 (right). Dashed box is magnified (bottom) showing separated channels. Scale bars: 50μm.

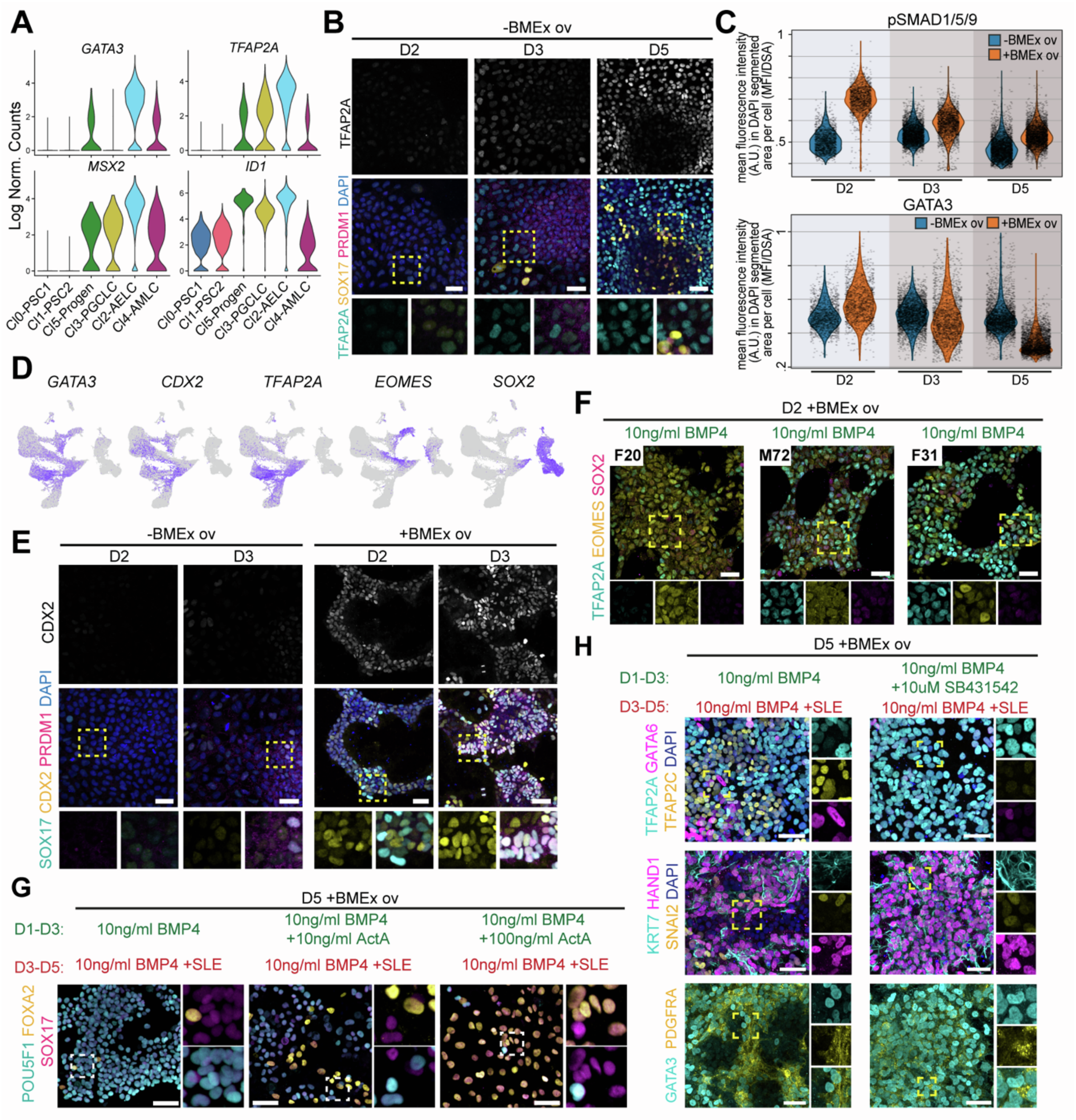

Figure S4. Characterization of D2-progenitors during hPGCLC differentiation with BMEx overlay. Related to Figure 6.

(A) Violin plots showing expression levels of selected genes of interest per cluster.

(B) Immunofluorescence for TFAP2A, SOX17, PRDM1 at D2, D3 and D5 without BMEx overlay in line M54. TFAP2A is shown on top as single channel. Dashed box is magnified (bellow) showing separated channels. Scale bars: 50µm.

(C) Violin plots depict the quantification of the images in Figure 6C as the mean fluorescence intensity in arbitrary units (A.U.) of pSMAD1/5/9 (top) and GATA3 (bottom) in DAPI segmented areas (normalized to 1) per cell.

(D) Expression of signature genes of hPGCLC-progenitors on the UMAP plot from Figure 3D.

(E) Immunofluorescence for SOX17, CDX2, PRDM1 at D2 and D3 with or without BMEx overlay. CDX2 is shown on top as single channel. Dashed box is magnified (bellow) showing separated channels. Scale bars: 50µm.

(F) Immunofluorescence for TFAP2A, EOMES, SOX2 at D2 with BMEx overlay in line F20, M72 and F31. Dashed box is magnified (bellow) showing separated channels. Scale bars: 50µm.

(G) Immunofluorescence for POU5F1, FOXA2, SOX17 at D5 with BMEx overlay in the indicated culture conditions. Dashed box is magnified (right) showing separated channels. Scale bars: 50μm.

(H) Immunofluorescence for TFAP2A, GATA6 and TFAP2C; KRT7, HAND1 and SNAI2; GATA3 and PDGFRA at D5 with BMEx overlay in the indicated culture conditions. Dashed box is magnified (right) showing separated channels. Scale bars: 50μm.
