# Supplementary material for: Efficient and scalable generation of primordial germ cells in 2D culture using basement membrane extract overlay
Source: Cell Rep Methods. 2023 May 23;3(6):100488. doi: 10.1016/j.crmeth.2023.100488 (PMC10326346; doi:10.1016/j.crmeth.2023.100488)
Supplement: Document S2. Article plus supplemental information [file mmc2.pdf]

Cell Reports
Methods

Article

Efficient and scalable generation of primordial germ cells in 2D culture using basement membrane extract overlay

Graphical abstract

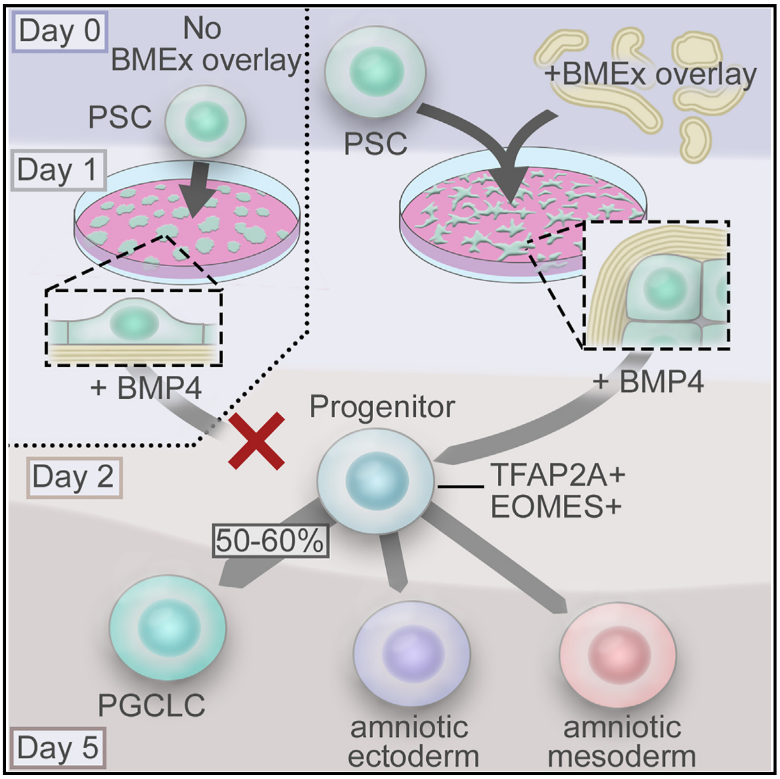

Highlights

- hPGCLCs are generated efficiently from hPSCs treated with BMP4 and BMEx overlay
- The BMEx overlay method is highly scalable, cost-effective, and simple to perform
- hPGCLCs differentiate together with amniotic ectoderm- and mesoderm-like cells
- BMEx overlay hPGCLCs express DDX4 and DAZL upon co-culture with fetal gonadal cells

Authors

Arend W. Overeem, Yolanda W. Chang, Ioannis Moustakas, ..., Hailiang Mei, Christian Freund, Susana M. Chuva de Sousa Lopes

Correspondence

lopes@lumc.nl

In brief

Overeem, Chang, et al. demonstrate an important role for extracellular matrix in human primordial germ cell-like cell (hPGCLC) differentiation from pluripotent stem cells. With combined treatment with basement membrane extract (BMEx) and BMP4, hPGCLCs form in standard 2D culture, yielding an efficient differentiation method that greatly facilitates *in vitro* gametogenesis research.

Overeem et al., 2023, Cell Reports Methods *3*, 100488
June 26, 2023 © 2023 The Authors.
https://doi.org/10.1016/j.crmeth.2023.100488

CellPress

Cell Reports Methods

CellPress
OPEN ACCESS

Article

Efficient and scalable generation of primordial germ cells in 2D culture using basement membrane extract overlay

Arend W. Overeem,[1,6] Yolanda W. Chang,[1,6] Ioannis Moustakas,[1,2] Celine M. Roelse,[1] Sanne Hillenius,[1] Talia Van Der Helm,[1] Valérie F. Van Der Schrier,[1] Manuel A.F.V. Gonçalves,[3] Hailiang Mei,[2] Christian Freund,[1,4] and Susana M. Chuva de Sousa Lopes[1,5,7,*]

[1]Department of Anatomy and Embryology, Leiden University Medical Center, 2333 ZC Leiden, the Netherlands
[2]Sequencing Analysis Support Core, Leiden University Medical Center, 2333 ZC Leiden, the Netherlands
[3]Department of Cell and Chemical Biology, Leiden University Medical Center, 2333 ZC Leiden, the Netherlands
[4]Leiden University Medical Center hiPSC Hotel, Leiden University Medical Centre, 2333 ZC Leiden, the Netherlands
[5]Department for Reproductive Medicine, Ghent University Hospital, 9000 Ghent, Belgium
[6]These authors contributed equally
[7]Lead contact
*Correspondence: lopes@lumc.nl
https://doi.org/10.1016/j.crmeth.2023.100488

MOTIVATION The ultimate goal of human *in vitro* gametogenesis is to generate functional gametes from human induced pluripotent stem cells (hiPSCs). However, current methods to differentiate human primordial germ cell-like cells (hPGCLCs) rely on embryoid body formation in a 96-well plate format and can be labor intensive and inefficient, and this severely limits the yield of hPGCLCs as well as any downstream applications. We present a highly robust hPGCLC differentiation method that is efficient, easy to perform, and compatible with any desired 2D culture surface.

SUMMARY

Current methods to generate human primordial germ cell-like cells (hPGCLCs) from human pluripotent stem cells (hPSCs) can be inefficient, and it is challenging to generate sufficient hPGCLCs to optimize *in vitro* gametogenesis. We present a differentiation method that uses diluted basement membrane extract (BMEx) and low BMP4 concentration to efficiently induce hPGCLC differentiation in scalable 2D cell culture. We show that BMEx overlay potentiated BMP/SMAD signaling, induced lumenogenesis, and increased expression of key hPGCLC-progenitor markers such as TFAP2A and EOMES. hPGCLCs that were generated using the BMEx overlay method were able to upregulate more mature germ cell markers, such as DAZL and DDX4, in human fetal ovary reconstitution culture. These findings highlight the importance of BMEx during hPGCLC differentiation and demonstrate the potential of the BMEx overlay method to interrogate the formation of PGCs and amnion in humans, as well as to investigate the next steps to achieve *in vitro* gametogenesis.

INTRODUCTION

In mammals, gametogenesis is a complex and long process that is initiated by the specification and lineage restriction of primordial germ cells (PGCs), the founding population of the gametes.[1] Recapitulating (female and male) gametogenesis *in vitro* would enable modeling of infertility-causing diseases and may ultimately lead to new assisted-reproduction techniques.

In mice, Bmp4 was identified as a crucial morphogen, inducing PGC specification in the posterior-proximal epiblast.[2] Acting through the intracellular factors Smad1/5/9, Bmp4 is able to upregulate *Tbxt* (*Brachyury* or *T*) as well as a specific gene regulatory network that includes *Prdm1*, *Prdm14*, and *Tfap2c*.[3] This knowledge has led to the recapitulation of mouse PGC-like cell (PGCLC) formation *in vitro* by exposure of mouse pluripotent stem cells (mPSCs) grown as embryoid bodies (EBs) to BMP4.[3] Subsequently, human PGCLCs (hPGCLCs) have been generated from human PSCs (hPSCs) using a similar approach,[4,5] highlighting the high degree of conservation between human and mouse regarding PGC specification but also uncovering differences in the specification mechanisms[6] as well as differences in their molecular signatures.[7]

The origin of PGCs in mice and humans *in vivo* may also differ. In mice, specified PGCs are located in the posterior-proximal

© 2023 The Authors.
This is an open access article under the CC BY license (http://creativecommons.org/licenses/by/4.0/).

epiblast at the base of the allantois shortly after the onset of gastrulation. Although it remains unknown when and where exactly PGC specification takes place in humans, in cynomolgus monkey embryos, PGCs were first observed in the amnion prior to gastrulation.[8] In contrast to mice and pigs, which undergo amniogenesis by folding, humans and non-human primates undergo amniogenesis by cavitation instead[9,10]; therefore, amnion and PGCs may share a similar origin in primates. In agreement, hPGCLCs share a common TFAP2A+ progenitor with amnion ectoderm-like cells in EB differentiation assays,[11] and hPGCLC formation has been demonstrated in an amniotic sac embryoid model.[12,13]

The most widely used directed differentiation protocols to derive hPGCLCs from hPSCs include EB aggregation and treatment with high concentrations of BMP4. However, while these EB-based methods were instrumental in understanding hPGCLC formation,[4,5] they can be characterized by low efficiency and high variability on a per-hPSC line basis.[14–16] Reported hPGCLC yields ranged from 5% to 60%, but for the majority of hPSC lines, hPGCLC differentiation efficiencies are below 10%. In addition, EB differentiation is low throughput, laborious, and requires harsh and stressful cell dissociation. As a result, efficient hPGCLC generation for high-throughput downstream experiments aiming at optimizing human gametogenesis *in vitro* remains challenging.

In this study, we have uncovered a critical role of the extracellular matrix (ECM) during hPGCLC differentiation from hPSCs in 2D culture. We show that the addition of basement membrane extract (BMEx) and BMP4 at a concentration as low as 10 ng/mL to an ordinary 2D cell culture format is sufficient to consistently generate hPGCLCs at high yields, ranging between 30% and 50%, within 5 days of differentiation. The hPGCLCs in this 2D system originated from a *TFAP2A+CDX2+GATA3+EOMES+* progenitor population that also gave rise to amniotic ectoderm-like and presumably amniotic mesoderm-like cells. Importantly, the presented hPGCLC differentiation method is highly scalable and cost effective, which will greatly facilitate progress achieving human *in vitro* gametogenesis (IVG).

RESULTS

Robust generation of hPGCLCs in 2D culture with BMEx overlay

The application of diluted BMEx on 2D plated hPSCs (BMEx overlay) has been shown to be important to induce lumen formation (lumenogenesis),[17] enabling that system to model aspects of early human embryogenesis.[18] As hPGCLCs formed readily in the amniotic sac embryoid model, which consisted of BMP4-treated hPSC spheres cultured on a microfluidic device,[12] we hypothesized that BMEx-supplemented culture may facilitate the generation of hPGCLCs in regular 2D culture formats. To test this, single-cell-passaged human induced PSCs (hiPSCs) were plated in mTeSR-plus medium supplemented with 2% BMEx (day 0) (Figure 1A). One day later (day 1), the medium was replaced with previously described hPGCLC induction medium[19] containing 200 ng/mL BMP4 as well as stem cell factor (SCF), leukemia inhibitory factor (LIF), and epidermal growth factor (EGF). The differentiation was carried out in hPGCLC-induction medium for 4 days (days 1–5), with the medium in the initial 2 days (days 1–3) supplemented with 2% BMEx (Figure 1A).

Pronounced morphological changes were observed when hiPSCs were differentiated with BMEx overlay (Figure 1B). In contrast to the flat colonies observed in the absence of BMEx overlay, tightly packed colonies were present with BMEx overlay. Immunofluorescence on day 5 of differentiation revealed a large number of ITGA6+POU5F1+SOX17+ hPGCLCs only in the BMEx overlay condition across three hPSC lines, M54, F99, and H1 (Figure 1C), in addition to the expression of other known PGC markers, such as TFAP2C, PDPN, PRDM1, and ALPL (Figure 1D). In agreement, flow cytometry analysis using PGC markers ITGA6 and EPCAM[5,20] revealed that the hPGCLC generation efficiency was about 50% in line M54, whereas basically no hPGCLCs were detected in the absence of BMEx overlay (Figure 1E), revealing a critical role for the cell-ECM interaction during hPGCLC differentiation.

Optimization of BMEx overlay differentiation method

The response of hPSCs to BMP4 signaling is highly dependent on both culture format and cell density.[21,22] The hPGCLC induction medium contained a high dose of BMP4 (200 ng/mL), which was optimized for EB-based methods. To establish the optimal BMP4 dosage in our 2D system, we tested different concentrations of BMP4 while removing SCF, LIF, and EGF from day 1 to 3 and reducing the concentration of BMP4 to 10 ng/mL from day 3 to 5 (Figure 2A). Strikingly, we observed comparable efficiencies to induce (ITGA6+EPCAM+) hPGCLCs with vastly reduced BMP4 concentrations in four independent hPSC lines (Figures 2B, S1A). Using immunofluorescence, we further confirmed an associated increase in POU5F1+SOX17+ hPGCLCs (Figure 2C).

In previous work using EB-based differentiation, we identified the lines F20 and M72 as inefficient hPGCLC-generating lines.[14] We observed the same in BMEx overlay differentiation, with F20 yielding 15% and M72 0.3%, respectively (Figure S1A). This suggested that variance in hPGCLC generation efficiency could be an inherent cell line property independent of the differentiation method used.[16]

It was previously demonstrated that activin A (ActA)/NODAL induced hPGCLC differentiation competency in hPSCs.[19] Moreover, the addition of a low dose of ActA together with BMP4 improved the specification of hPGCLCs in micropatterned colonies.[23] Hence, we tested whether exogenous ActA could increase induction of hPGCLCs in our system (Figure 2D). We observed that simultaneous treatment with BMP4 and ActA from day 1 to 3 lowered the hPGCLCs' yield in all tested concentrations compared with treatment with BMP4 alone (Figure 2D); shortening the ActA treatment to 1–2 days gave a similarly poor outcome (Figure S1B). Interestingly, inhibiting endogenous transforming growth factor β (TGF-β)/ActA signaling by blocking the receptor type I (ALK4/ACVR1B, ALK5/TGFBR1, ALK7/ACVR1C) using SB431542 reduced hPGCLC formation (Figures 2D, S1B).

BMEx is a biologically complex product of animal origin that is highly prone to variabilities between batches and manufacturers. To determine the robustness of using BMEx for hPGCLC differentiation, we tested multiple lots of three commercially available stem cell-grade BMEx products. Geltrex and Cultrex consistently induced hPGCLC formation with about 50% efficiency

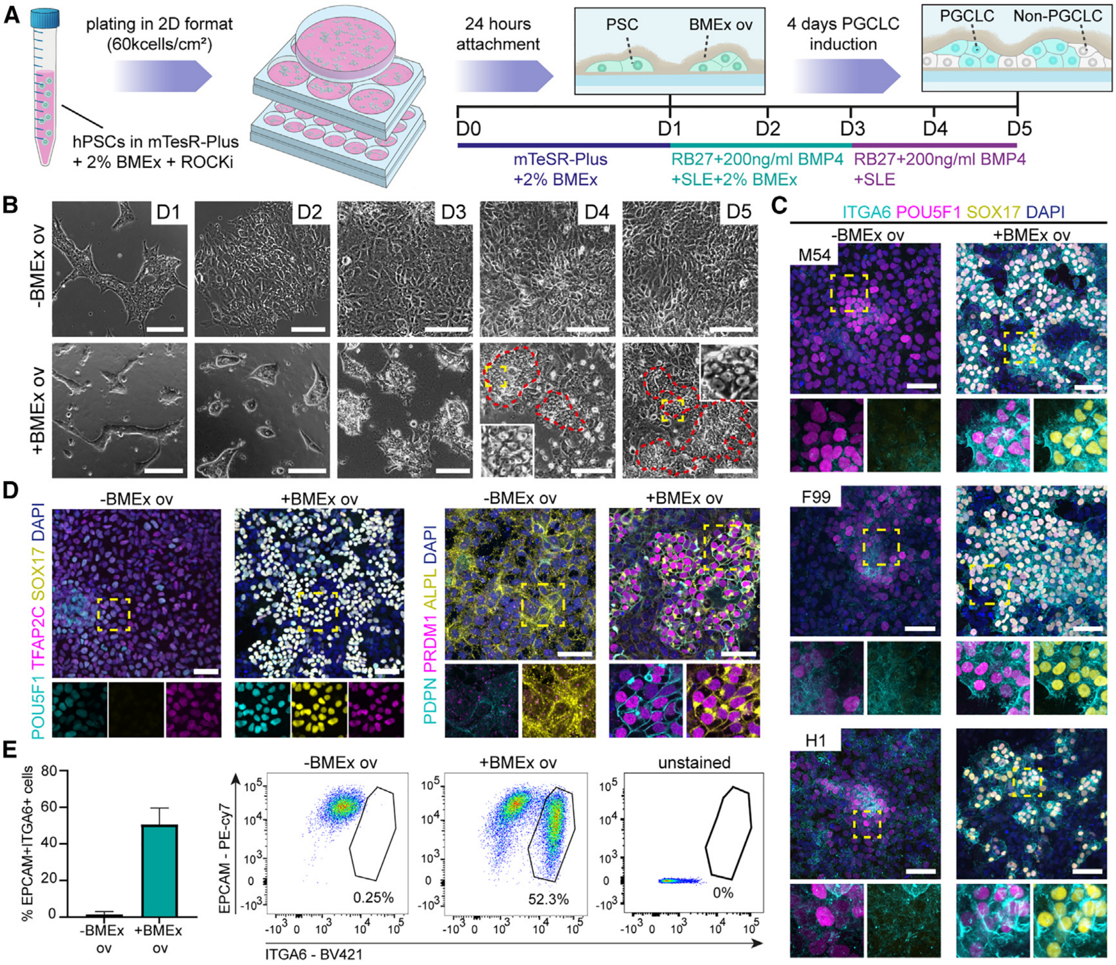

Figure 1. Robust generation of hPGCLCs in 2D culture using BMEx overlay method

(A) Schematic representation of the BMEx overlay method. SLE, SCF, LIF, EGF; RB27, Advanced RPMI 1640 + B27.

(B) Bright-field images of −BMEx and +BMEx overlay during differentiation (days 1–5). Red lines depict compacted clumps of cells. Yellow dashed box is magnified. Scale bars: 100 μm.

(C) Immunofluorescence for ITGA6, POU5F1, and SOX17 at day 5 with or without BMEx overlay in lines M54, F99, and H1. Dashed box is magnified (bottom), showing separated channels. Scale bars: 50 μm.

(D) Immunofluorescence for POU5F1, TFAP2C, and SOX17 (left) and PDPN, PRDM1, and ALPL (right) at day 5 with or without BMEx overlay in line M54. Dashed box is magnified (bottom), showing separated channels. Scale bars: 50 μm.

(E) Bar graph (left) showing mean percentage of double EPCAM+ITGA6+ cells (n = 3) at day 5 with or without BMEx overlay in line M54 analyzed by FACS; error bars represent mean ± SD; and representative FACS plots showing the gating used (right).

(Figures 2E and 2F). Surprisingly, Matrigel only had a minor hPGCLC-inducing effect (Figures 2E and 2F). This outcome was not due to differences in total protein concentrations (Figure S1C). Moreover, increasing the percentage of Matrigel during differentiation to up to 3.5% had no effect on differentiation efficiency (Figure S1D).

Efficient PGCLC differentiation is accompanied by parallel induction of amniotic ectoderm-like and mesoderm-like cells

To identify the cell types present in our BMEx overlay model during differentiation (2% BMEx overlay from day 0 to 3 and 10 ng/mL BMP4 from day 1 to 5), we performed single-cell transcriptomics of two PGCLC-efficient lines (M54 and F99) and two PGCLC-inefficient lines (F20 and M72) at days 0, 2, and 5 (Figures 3A and S2A).

Visualization by uniform manifold approximation and projection (UMAP) revealed the presence of six clusters (Cl0–Cl5) (Figure 3A). The top 12 most differentially expressed genes (DEGs) (based on the average log2 fold change [avg_log2FC]) indicated that Cl0–Cl1 consisted of day 0 PSCs expressing high levels of *SOX2*; Cl5 consisted of day 2 progenitor cells expressing high levels of BMP signaling target genes *ID1* and *ID3*; Cl3 corresponded to PGCLCs expressing *NANOS3* and *PDPN*; Cl2 corresponded to human amniotic ectoderm-like cells (AELCs) expressing *ISL1*, *GATA3*, *TFAP2A*, and *KRT7*; and Cl4

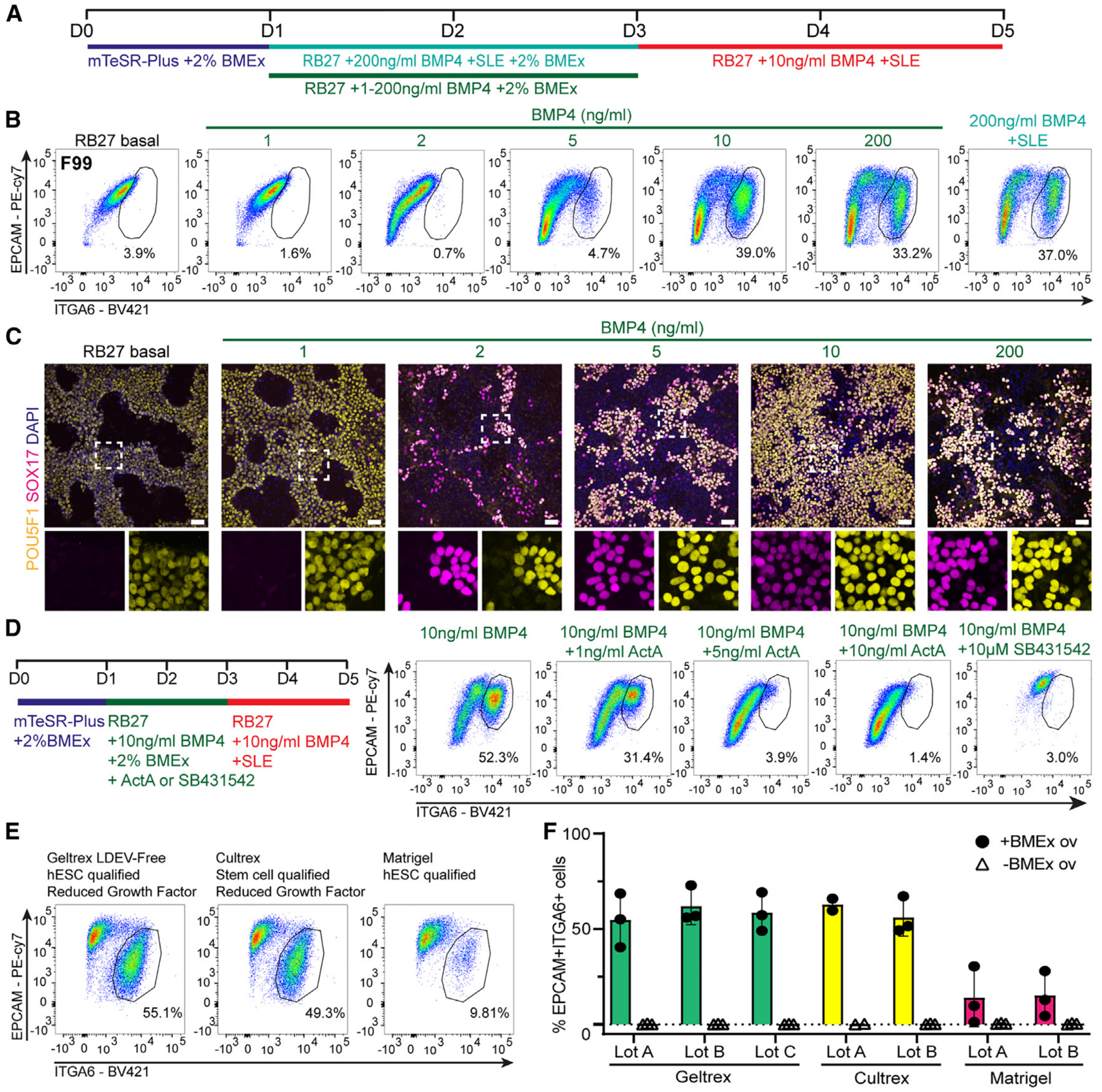

Figure 2. Optimization of 2D BMEx overlay method

(A) Experimental scheme depicting the different conditions tested in (B) and (C).

(B) FACS plots depicting the percentage of double EPCAM+ITGA6+ cells at day 5 in line F99 to test different BMP4 concentrations.

(C) Immunofluorescence for POU5F1 and SOX17 at day 5 in line F99 to test different BMP4 concentrations. Dashed box is magnified (bottom), showing separated channels. Scale bars: 50 μm.

(D) Experimental scheme depicting different conditions tested (left) and the associated FACS plots (right) depicting the percentage of double EPCAM+ITGA6+ cells at day 5 in line F99.

(E) Representative FACS plots showing percentages of double EPCAM+ITGA6+ cells at day 5 generated using Geltrex, Cultrex, and Matrigel overlay in line M54.

(F) Bar graph showing mean percentages of double EPCAM+ITGA6+ cells at day 5 generated using different brands and batches of BMEx, analyzed by FACS in line M54; error bars represent mean ± SD.

See also Figure S1.

corresponded to human extraembryonic/amniotic mesoderm-like cells (AMLCs) expressing *TMEM88*, *BMP4*, *COL3A1*, and *COL6A3* (Figure 3B).

We further confirmed cell type identity by the expression of known marker genes[10,11,24]: PGCLC (Cl3) and PSCs (Cl0–Cl1) expressed high levels of *POU5F1* and *NANOG*, but *PRDM1* and *SOX17* were exclusively expressed by PGCLCs (Figure 3C); AELCs (Cl2) and AMLCs (Cl4) shared high expression of *HAND1*, but only AMLCs expressed high levels of *GATA6*, *PDGFRA*, and *FOXF1*, whereas many cells in AELCs expressed *ISL1*, *TFAP2A*, *VTCN1*, and *IGFBP3* (Figures 3C, S2B); and a small subset of cells in Cl2 expressed key endoderm markers *FOXA2*, *HNF1B*, *HNF4A*, and *SOX17*,[24] presumably too small to result in a separate cluster (Figure S2C).

As expected, Cl3 (PGCLCs) was comprised almost exclusively of cells derived from the efficient PGCLC-generating PSC lines

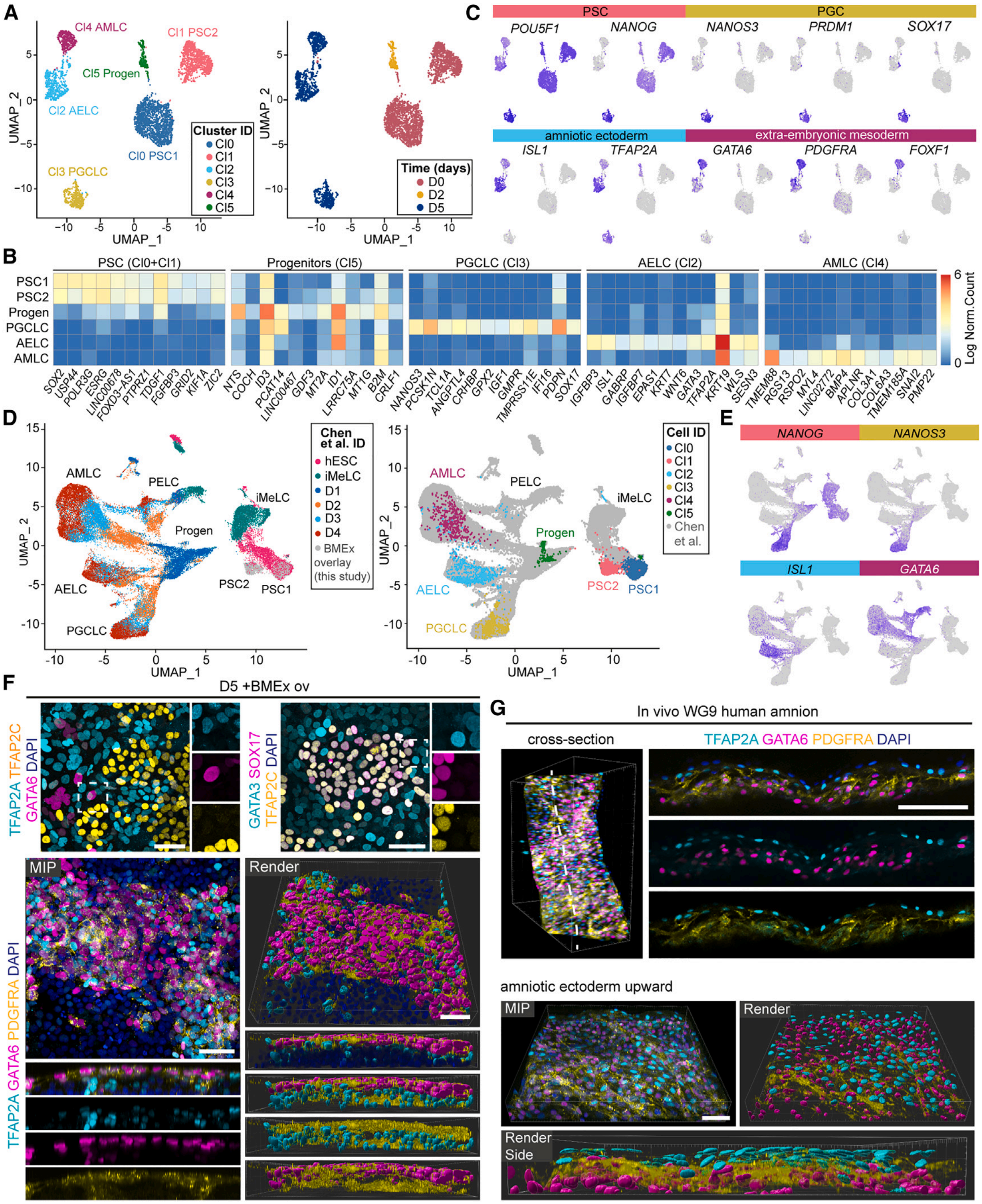

Figure 3. BMEx overlay differentiation promotes differentiation to hPGCLCs alongside amniotic ectoderm- and mesoderm-like cells

(A) Uniform manifold approximation and projection (UMAP) plots showing cluster identification (ID) (left) and time period in days (right) using single-cell transcriptomics of several hPSC lines undergoing differentiation with BMEx overlay.

(B) Heatmap showing expression levels of the top 12 DEGs of each cluster.

(C) Expression of signature genes of cell types of interest on the UMAP plot from (A).

(D) UMAP showing integrated single-cell transcriptomics data from EB differentiation method (UCLA2 from Chen et al.[11]) and BMEx overlay method, highlighting the cells of EB differentiation method (left) and the BMEx overlay method (right).

(E) Expression of signature genes of cell types of interest on the UMAP plot from (D).

(F) Immunofluorescence for TFAP2A, TFAP2C, and GATA6 (top left); GATA3, SOX17, and TFAP2C (top right); and TFAP2A, GATA6, and PDGFRA (bottom) at day 5 with BMEx overlay. In the top panels, the dashed box is magnified (right), showing separated channels. The bottom panels depict a maximum intensity projection (MIP) and render image with a digital cross section showing separated channels (bottom part). Scale bars: 50 μm.

(legend continued on next page)

M54 and F99 and a very small fraction from F20 (Figure S2A). Intriguingly, at day 0, the hiPSCs formed two separate clusters: Cl0 included cells from M54, F99, and F20, whereas Cl1 included cells from M72 and F20 (Figure S2A). This suggested that F20 consisted of two subpopulations with distinct transcriptomes, one similar to M72 and the other to M54 and F99. This is consistent with the observation that, unlike M72, F20 could generate 15% PGCLCs (Figure S1A). We performed differential expression analysis between Cl0 and Cl1 and observed that Cl1 showed higher levels of *UTF1*, *NODAL*, *PRAC1*, and *SIX3*, whereas Cl0 cells expressed *SFRP1*, *PCLAF*, *RAB17*, and *TAGLN* (Figure S2D). We speculate that these genes, in particular *NODAL*, may be used as potential markers to distinguish efficient from inefficient hPGCLC-generating hPSCs, although characterization of additional hPSC lines will be required to verify this.

To compare the developmental timeline and cell types generated using our BMEx overlay method with the "conventional" EB differentiation method, we merged our single-cell dataset with the single-cell dataset generated by Chen and colleagues[11] using the "conventional" EB differentiation method[5] (Figures 3D, S2E, and S2F). The molecular signatures were largely similar, and the three endpoint cell types PGCLCs, AELCs, and AMLCs from the BMEx overlay method mapped on to the PGCLC, amnion-like cells and extraembryonic mesenchyme (EXMC) from the Chen dataset. A small population of human endoderm-like cells (part of Cl2) now formed an independent cluster together with cells previously identified as primitive endoderm-like cells (Figures 3D and S2F). Hence, we demonstrated that despite the different culture formats and pre-treatment step, both differentiation methods generate the same cell types, and in both methods, PGCLCs are formed alongside amnion-like cells.

The observation that PGCLCs arise alongside amniotic cells has also been made when using the amniotic sac embryoid system (μPASE).[13] To compare the cell types generated by BMEx overlay differentiation and the μPASE method, we merged our data with single-cell transcriptomics data generated by Zheng and colleagues[13] (Figures S2G and S2H). As expected, our PGCLCs clustered together with the PGCLCs in the μPASE, expressing *NANOS3* and *PRDM1* (Figure S2H). Our AELCs clustered together with amniotic ectoderm-like cells named AMLCs (AMLC2s)in the μPASE, expressing *TFAP2A*, *ISL1*, and *GABRP* (Figure S2H). Finally, our AMLCs clustered together with mesoderm-like cells (MeLC1s) in the μPASE, expressing *GATA6*, *PDGFRA*, *FOXF1*, and *SNAI2* (Figure S2H).

Next, we validated by immunofluorescence the three main cell types present in culture at day 5: TFAP2C+/SOX17+ PGCLCs, TPAP2A+/GATA3+/SNAI2+/KRT7+ AELCs, and GATA6+/PDGFRA+ AMLCs (Figures 3F and S2I). Interestingly, without BMEx overlay, the culture consisted mostly of AELCs, expressing TFAP2A, GATA3, KRT7, and HAND1, and a very small minority of GATA6+/PDGFRA+ AMLCs (Figure S2J). Finally, using human amnion from 9 weeks of gestation (WG9), we confirmed by whole-mount immunofluorescence the expression of TFAP2A in the amniotic ectoderm and GATA6 and PDGFRA in amniotic mesoderm (Figure 3G).

The transcriptome of PGCLCs is similar to that of Carnegie stage 7 human PGCs

To compare the transcriptome of our PGCLCs and amnion-like cells with that of their *in vivo* counterparts, we merged our single-cell RNA sequencing (RNA-seq) dataset with an available single-cell RNA-seq dataset from a Carnegie stage 7 (CS7) human embryo.[24] UMAP visualization of the merged datasets showed that the cell types *in vitro* clustered with the corresponding *in vivo* counterparts (Figure 4A). The day 0 PSCs clustered with epiblast cells, whilst PGCLCs clustered with PGCs marked by *NANOS3* and *POU5F1* (Figure 4B). Moreover, AELCs clustered with amniotic ectoderm, both expressing *TFAP2A*, *ISL1*, *KRT7*, and *GATA3* (Figures 4A and 4B), whereas AMLCs clustered with advanced mesoderm, expressing *GATA6* and *PDFGRA* (Figures 4A and 4B). Since amniotic ectoderm cells are present in the dataset of the human embryo, but the extraembryonic mesoderm cells covering the amniotic ectoderm are missing from the annotation, it is likely that the authors annotated those (and perhaps the related mesodermal cells forming the connecting stalk) as advanced mesoderm.

Next, we compared the PGCLCs generated with the BMEx overlay method with more mature human fetal germ cells (FGCs) by merging our dataset with available single-cell RNA-seq data from first and second trimester human fetal gonads.[25] Visualization by UMAP showed that PGCLCs clustered with migratory and mitotic FGCs, which expressed PGC markers *POU5F1* and *NANOS3* (Figures 4C and 4D). In contrast to FGCs, PGCLCs did not express more mature FGC markers such as *DDX4*, *DAZL*, and *SYCP3* (Figure 4D), confirming that PGCLCs were similar to pre-migratory PGCs. Comparing the expression of a set of known PGC and germ cell markers between *in-vitro*-generated PGCLCs, CS7 PGCs, and FGCs indicated that PGCLCs, regardless of the differentiation method used, were most similar to CS7 PGCs (Figure 4E). Strikingly, PGCLCs showed lower expression of *KIT*, *DMRT1*, and *DPPA3* than CS7 PGCs (Figure 4E), suggesting that PGCLCs may be less mature than CS7 PGCs.

Lumenogenesis and PGCLC differentiation are independent events

A particular feature of BMEx overlay culture is the formation of lumen-containing structures.[17] Since we observed distinctive morphology resembling tube/lumen structures in the BMEx overlay method, we investigated whether lumenogenesis is linked to the differentiation of PGCLCs. We were able to detect laminin deposition on top of formed luminal structures at day 2 of differentiation with BMEx overlay (Figure 5A). By contrast, in the absence of the BMEx overlay, cells remained as a single-cell

(G) Whole-mount immunofluorescence for TFAP2A, GATA6, and PDGFRA in human WG9 amnion. In the top panels, a digital cross section (right) shows separated channels. The bottom panels depict a MIP and render image, also shown from the side (bottom). Scale bars: 50 μm. AELC, amniotic ectoderm-like cell; AMLC, amniotic mesoderm-like cell; iMeLCs, incipient mesoderm-like cells; PELC, primitive endoderm-like cell; Progen, progenitor cells.
See also Figure S2.

CellPress
OPEN ACCESS

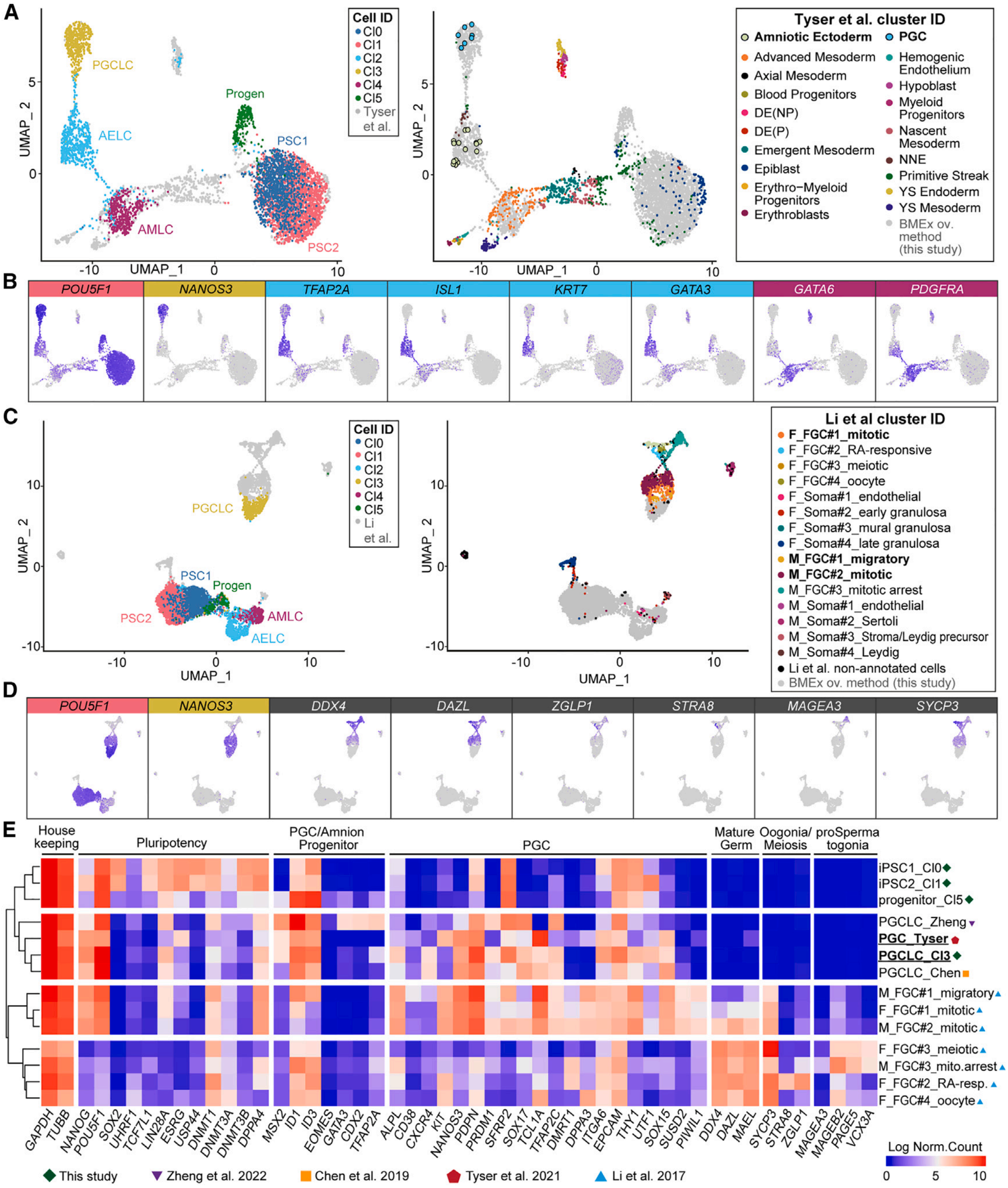

Figure 4. Comparison of *in-vitro*-generated hPGCLCs with *in vivo* counterparts

(A) UMAP showing integrated single-cell transcriptomics data from Carnegie stage 7 human embryo and BMEx overlay method, highlighting BMEx overlay method (left) and human embryo cells (right).

(B) Expression of signature genes of cell types of interest on the UMAP plot from (A).

(C) UMAP showing integrated single-cell transcriptomics data from first and second trimester human fetal gonads and BMEx overlay method, highlighting BMEx overlay method (left) and human fetal gonads (right).

(D) Expression of signature genes of cell types of interest on the UMAP plot from (C).

(E) Heatmap with hierarchical clustering showing expression level of selected genes in the BMEx overlay and germ cell clusters from all analyzed RNA-seq datasets.

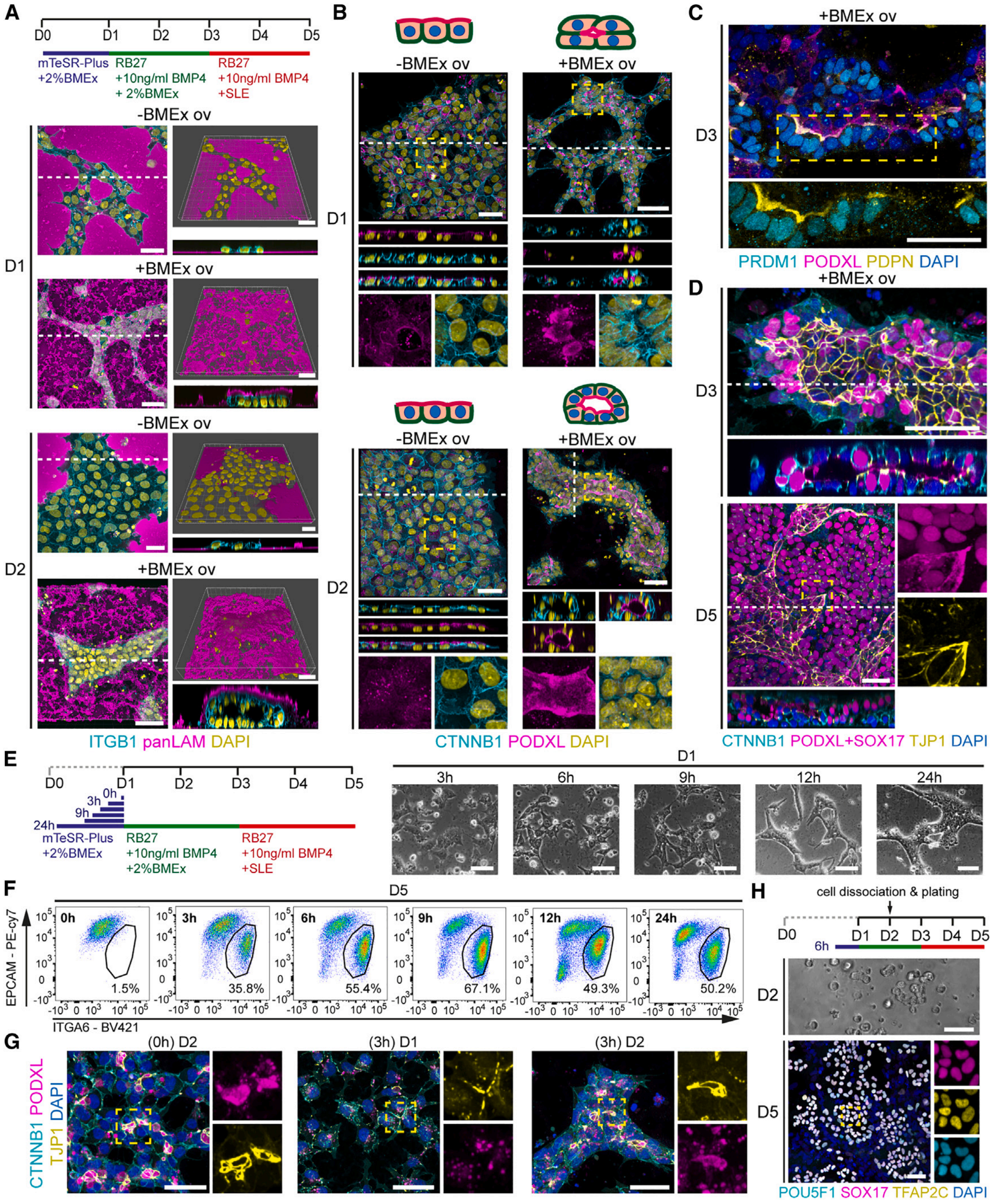

Figure 5. Differentiation of hPGCLCs is not coupled to lumenogenesis but depends on BMEx overlay

(A) Experimental scheme depicting the conditions used (top) and immunofluorescence for ITGB1 and panLAM as MIP (left) and surface render image (top right) at days 1 and 2, with or without BMEx overlay. Dashed line in left panel shows the level of the digital cross section (bottom right). Scale bars: 50 μm.

(B) Immunofluorescence for CTNNB1 and PODXL at days 1 and 2 with or without BMEx overlay. White dashed line shows the level of the digital cross section (middle panels), and the yellow dashed box is magnified (bottom), showing separated channels. Scale bars: 50 μm.

(C) Immunofluorescence for PRDM1, PODXL, and PDPN at day 3 with BMEx overlay. Dashed box is magnified (bottom), showing separated channels. Scale bar: 50 μm.

(D) Immunofluorescence for CTNNB1, PODXL+SOX17, and TJP1 at days 3 and 5 with BMEx overlay. White dashed line shows the level of the digital cross section (bottom), and the yellow dashed box is magnified (right), showing separated channels. Scale bars: 50 μm.

(E) Experimental scheme depicting the different conditions tested in (F) and (G) (left) and associated bright-field images at day 1 with BMEx overlay. Scale bars: 50 μm.

(legend continued on next page)

layer (Figure 5A). Moreover, we observed the expression of basal-lateral markers ITGB1 and CTNNB1, apical marker PODXL, and tight-junction marker TJP1 (Figures 5A, 5B, and S3A), confirming lumenogenesis at days 1–2. At day 3, the lumen expanded, and SOX17+/PRDM1+/PDPN+ PGCLCs were visible adjacent to the lumen (Figures 5C and 5D). At day 5, the lumens lost structural integrity, and a large number of SOX17+ PGCLCs could be observed (Figures 5D and S3B). In contrast to TPAP2A+ AELCs that expressed a clear rim of TJP1, SOX17+ PGCLCs only showed a focal accumulation of TJP1 (Figure S3B).

Next, we varied the period of the initial plating step (mTesR-plus +2% BMEx) from 24 to 0 h (cells plated directly in RB27 + 10 ng/mL BMP4 + 2% BMEx) (Figures 5E–5G) to investigate whether PGCLC differentiation depended on the timing of lumen formation. Although the initial plating step was necessary to obtain PGCLC differentiation, a 3 h plating step was sufficient to obtain robust differentiation to PGCLCs using two different lines, M54 (Figure 5F) and F99 (Figure S3C). Interestingly, independently of the duration of the initial plating step (between 0 and 24 h), small lumens marked by PODXL+TJP1+ apical membrane domains were observed by day 2 (Figure 5G). In the absence of BMEx overlay, we observed cellular polarization with the formation of a clear TJP1+ apical rim but no lumen formation (Figure S3D).

To further test whether lumen maintenance at day 2 was necessary for PGCLC differentiation, we disrupted the lumens at day 2 by dissociating and replating the cells, followed by analysis at day 5 (Figure 5H). Despite the disruption of lumens at day 2, immunofluorescence revealed the formation of TFAP2C+/SOX17+/POU5F1+ PGCLCs (Figure 5H). In conclusion, lumenogenesis and PGCLC differentiation appeared to be two independent events, and only exposure to BMEx (for a period as short as 3 h) prior to BMP4+BMEx treatment (for 2 days) was essential for PGCLC differentiation.

BMEx overlay potentiates BMP4 signaling in PGCLC progenitors at day 2

We performed differential expression analysis between the PSCs at day 0 (Cl0 and Cl1) and the progenitor population at day 2 (Cl5) and observed that from day 0 to 2, *DPPA4* and *SOX2* were downregulated, whereas many BMP responsive genes such as *ID1*, *ID3*, *GATA3*, *TFAP2A*, and *MSX2* were upregulated (Figures 6A and S4A). In addition, TFAP2A was expressed in the progenitor cells at days 2–3 but not in PRDM1+/SOX17+ PGCLCs at day 5 (Figure 6B). Interestingly, in the absence of BMEx overlay, TFAP2A was basically absent at day 2 (Figure S4B), whereas GATA3 showed comparable levels with or without BMEx overlay at days 2–3 (Figures 6C and S4C).

To test whether BMP4 signaling was influenced by BMEx overlay, we examined the levels of phosphorylated (p)SMAD1/5/9 (Figures 6C, S4C). Even though both culture conditions (with and without BMEx overlay) contained 10 ng/mL BMP4, the fluorescence intensity of nuclear pSMAD1/5/9 was higher in the presence of BMEx overlay, in particular at day 2 (Figures 6C and S4C).

In addition to *TFAP2A* and *GATA3*, *CDX2* and *EOMES* were also identified as markers of PGCLC progenitors in EB differentiation[11] and the BMEx overlay method (Figure S4D). In agreement, similarly to TFAP2A, both CDX2 and EOMES were upregulated at days 2–3 only in the presence of BMEx overlay (Figures 6D, S4E, and S4F). *EOMES* was previously shown to be activated by ActA/NODAL signaling during PGCLC differentiation and to be essential for initiating the PGCLC transcriptional network.[6,19] However, we observed that the addition of exogenous ActA was detrimental for PGCLC differentiation in the BMEx overlay method (Figure 2D). To understand this discrepancy, we quantified the expression of EOMES and TFAP2A in the common progenitor population at day 2 in the presence or absence of BMEx overlay and after treatment with 10 ng/mL ActA or inhibition of endogenous TGF-β/ActA signaling using 10 μM SB431452 (Figures 6D and 6E).

Compared with the absence of BMEx overlay, the day 2 progenitors cultured with BMEx overlay upregulated both TFAP2A and EOMES and downregulated SOX2 in line F99 (Figure 6D) and in lines F20, M72, and F31 (Figure S4F). When treated with 10 ng/mL BMP4 and 10 ng/mL ActA in the presence of BMEx overlay, day 2 progenitors upregulated EOMES considerably, whereas inhibition of endogenous TGF-β/ActA signaling blocked EOMES expression (Figures 6D and 6E), indicating that EOMES is strongly regulated by ActA signaling in our culture system. Interestingly, treatment with a combination of BMP4 and ActA from day 1 to 3 with BMEx overlay resulted at day 5 in the induction of SOX17+-FOXA2+ cells (Figure S4G), presumably endoderm, which is consistent with the role of ActA and its target EOMES in endoderm differentiation.[26,27] Blocking endogenous TGF-β/ActA signaling from day 1 to 3, on the other hand, resulted in generation of mainly amniotic ectoderm cells at day 5, expressing markers such as TFAP2A, KRT7, HAND1, and GATA3 (Figure S4H).

In conclusion, in our optimized PGCLC differentiation method, the addition of BMEx overlay between days 0 and 3 resulted in faster downregulation of SOX2, increased pSMAD1/5/9 signaling, and increased expression of TFAP2A, CDX2, and EOMES (Figure 6F). This led to the formation of nascent PGCLCs at day 3, with downregulation of TFAP2A and CDX2 and upregulation of *NANOS3* by day 5, which make up about 50% of the cells in culture, alongside amniotic ectoderm- and mesoderm-like cells (Figure 6F).

In vitro maturation of hPGCLCs by co-culturing with human fetal ovary cells

The transcriptome of hPGCLCs resembles pre-migratory PGCs that still lack expression of *DDX4* and *DAZL* (Figure 4E). Previous studies have shown that hPGCLCs co-cultured with mouse fetal

(F) FACS plots depicting the percentage of double EPCAM+ITGA6+ cells at day 5 in line M54 to test different priming periods.
(G) Immunofluorescence for CTNNB1, PODXL, and TJP1 at days 1 and 2 to test different priming periods. Dashed box is magnified (right), showing separated channels. Scale bars: 30 μm.
(H) Experimental scheme depicting the conditions used to disrupt the lumens at day 2 (top), associated bright-field image at day 2 after dissociation, and immunofluorescence for POU5F1, SOX17, and TFAP2C at day 5. Dashed box is magnified (right), showing separated channels. Scale bars: 50 μm.
See also Figure S3.

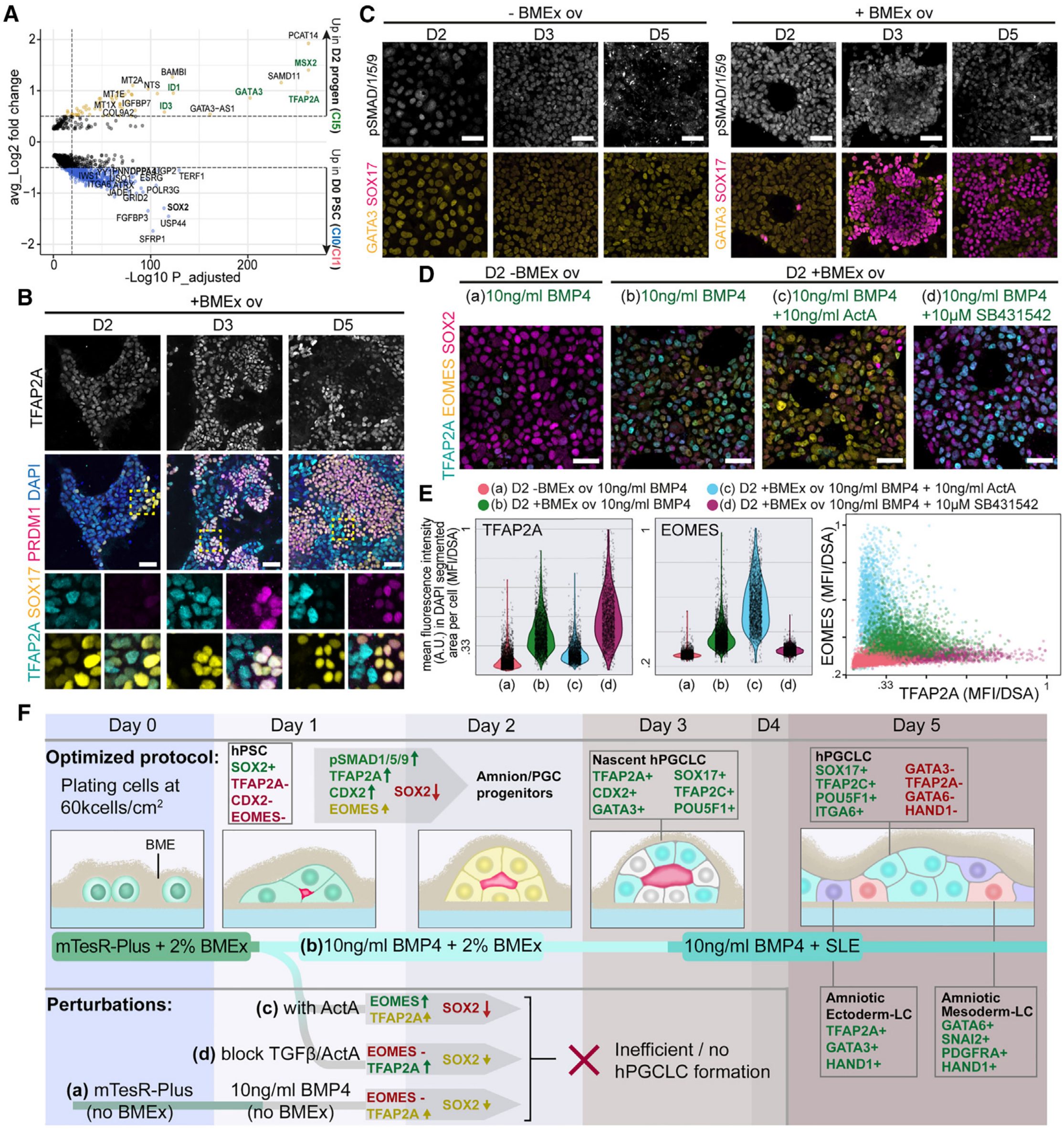

Figure 6. BMEx overlay potentiates BMP4 signaling and increases expression of critical PGC specification factors

(A) Volcano plot showing DEGs between hPSCs at day 0 and 2-differentiated progenitors with BMEx overlay.

(B) Immunofluorescence for TFAP2A, SOX17, and PRDM1 at days 2, 3, and 5 with BMEx overlay in line M54. TFAP2A is shown on top as a single channel. Dashed box is magnified (below), showing separate channels. Scale bars: 50 μm.

(C) Immunofluorescence for pSMAD1/5/9, GATA3, and SOX17 at days 2, 3, and 5 with or without BMEx overlay in line M54. pSMAD1/5/9 is shown on top as a single channel. Scale bars: 50 μm.

(D) Immunofluorescence for TFAP2A, EOMES, and SOX2 in line F99 at day 2 without (a) or with (b) BMEx overlay or with addition of 10 ng/mL activin A (C) or 10 μM SB431542 (D). Scale bars: 50 μm.

(E) Violin plots depict the quantification of the images in (D) as the mean fluorescence intensity in arbitrary units (A.U.) of TFAP2A (left) and EOMES (middle) in DAPI segmented areas (normalized to 1) per cell at day 2. The correlation between these two values per cell per condition was visualized in a scatterplot (right).

(F) Cartoon summarizing the hPGCLC differentiation progression in the BMEx overlay method as well as the perturbations tested (from D and E), with key analyzed markers depicted.

See also Figure S4.

ovary or testis cells resulted in DDX4+SYCP3+ oogonia-like cells or DDX4+MAGEA3+ prospermatogonia-like cells, respectively, after 120 days of culture.[28,29] To investigate whether hPGCLCs generated with the BMEx overlay method have the potential to mature further, we co-cultured hPGCLCs with cells isolated from human fetal ovaries (Figure 7A). To track the hPGCLCs in the co-culture, we generated a female POU5F1::EGFP reporter hiPSC line by fusing EGFP to the C terminus of POU5F1 using

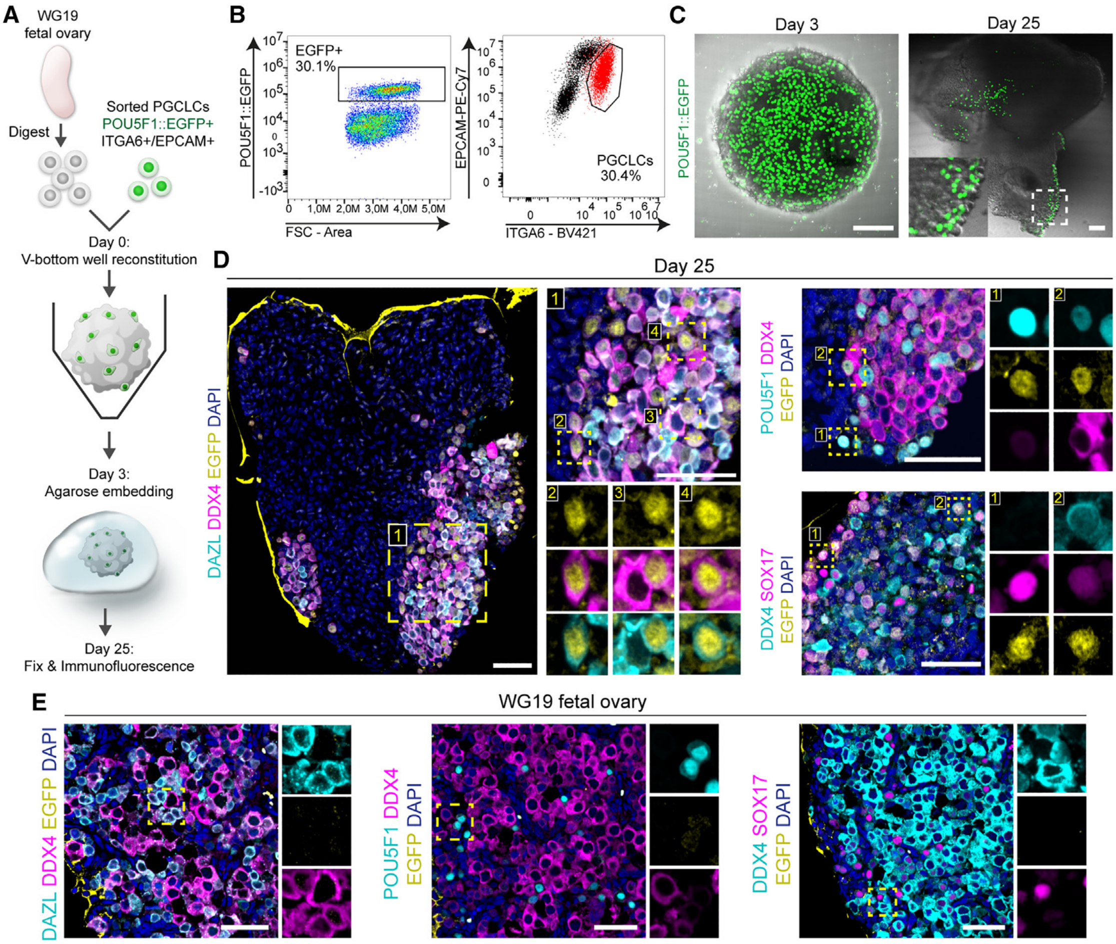

Figure 7. ***In vitro*** **maturation of hPGCLCs by co-culture with human fetal ovary cells**

(A) Experimental schematics depicting the workflow for the reconstituting human fetal ovarian cells with hPGCLCs.

(B) Representative FACS plot showing percentages of POU5F1::EGFP+ cells (left) and EPCAM+ITGA6+ cells (right), with POU5F1::EGFP+ cells highlighted in red and POU5F1::EGFP− cells in black.

(C) Live images showing POU5F1::EGFP in human fetal ovary/hPGCLC aggregates at days 3 and 25 of culture. Scale bars: 100 μm.

(D) Immunofluorescence for EGFP, DDX4, and DAZL (left); EGFP, DDX4, and POU5F1 (top right); and EGFP, DDX4, and SOX17 (bottom right) in human fetal ovary/hPGCLC aggregates at day 25. Dashed box is magnified, showing separated channels. Scale bars: 50 μm.

(E) Immunofluorescence for EGFP, DDX4, and DAZL (left); EGFP, DDX4, and POU5F1 (middle); and EGFP, DDX4, and SOX17 (right) in WG19 human fetal ovary. Dashed box is magnified, showing separated channels. Scale bars: 50 μm.

in *trans* paired nicking genome editing.[30] This method is based on CRISPR-Cas9 nickases instead of nucleases and allows for the isolation of POU5F1::EGFP-tagged hiPSCs while minimizing endogenous *POU5F1* disruption[31] and off-target *EGFP* tag insertions.[32]

Next, we differentiated the POU5F1::EGFP reporter line into hPGCLCs using the BMEx overlay method and used fluorescence-activated cell sorting (FACS) to isolate POU5F1EGFP+/ITGA6+/EPCAM+ hPGCLCs from day 5 culture (Figure 7B). Subsequently, we aggregated the POU5F1::EGFP+/ITGA6+/EPCAM+ hPGCLCs with dissociated WG19 fetal ovarian cells and cultured them for 3 days in ultra-low attachment 96-well V bottom plates before embedding in agarose droplets (Figure 7A). Live imaging of the aggregates at day 3 showed that EGFP+ hPGCLCs spread evenly in the aggregates (Figure 7C). At day 25 of culture, as the aggregate increased in size, EGFP+ hPGCLCs were still present in the aggregate but concentrated in specific regions (Figure 7C) and expressed hPGC markers such as POU5F1 and SOX17 (Figure 7D). Moreover, some EGFP+ cells started expressing more mature germ cell markers, such as DDX4 and DAZL (Figure 7D), similar to WG19 FGCs *in vivo* (Figure 7E). In conclusion, we have shown that hPGCLCs generated with the BMEx overlay method are capable of maturing to DDX4+/DAZL+ germ cells when co-cultured with human fetal ovarian cells.

DISCUSSION

The current methods to generate hPGCLCs *in vitro* have drawbacks regarding efficiency and scalability. As a consequence,

progress regarding the differentiation of hPGCLCs into more mature germ cells *in vitro* has been hampered. We report a new hPGCLC differentiation method that is efficient, simple, and cost effective in a highly scalable 2D format. This new method will contribute to accelerate the progression of human IVG research, and as such, we were able to demonstrate that hPGCLCs generated using the BMEx overlay method matured into DDX4+/DAZL+ germ cells when co-cultured with fetal ovary cells. Compared with a previous study that used reconstitution with mouse somatic niche to mature hPGCLCs in 77 days,[29] we observed upregulation of DDX4 in hPGCLCs by day 25 after reconstitution with human fetal ovarian cells.

There have been several previous reports on the generation of hPGCLCs in 2D culture. Differentiation of hiPSCs grown as small micropatterned colonies enabled generation of hPGCLCs with up to 70% efficiency.[23] Interestingly, substantial changes in cell-cell interaction and cell morphology took place in both the micropatterned and our BMEx overlay cultures compared with regular 2D culture. As such, these changes might be linked to a shared mechanism that enabled hPGCLC differentiation in both systems. While both methods showed high hPGCLC differentiation efficiency, the BMEx overlay method does not require manufacture of a specialized cell culture surface and is therefore less technically demanding. A second 2D hPGCLC differentiation method relies on WNT inhibition to improve hPGCLC specification, achieving around 20%–30% differentiation efficiency.[33]

The recent single-cell transcriptomics dataset of a single gastrulating CS7 human embryo, containing both amnion and hPGCs, is a tremendous resource for comparing *in-vitro*-differentiated cells with their *in vivo* counterparts.[10,24] We were able to verify that PGCLCs and AELCs showed similar transcriptomes to the PGCs and amniotic ectoderm in the human embryo, respectively. Interestingly, the AMLCs clustered together with PDGFRA+/GATA6+ mesodermal cells, annotated as advanced mesoderm in the Tyser dataset. Since the amniotic mesoderm annotation is missing in the Tyser dataset, but considering that that cell population must be present, as those cells are in close contact with the (annotated) amniotic ectoderm, we suggest that cell type may have been labeled as advanced mesoderm. In support of this, we showed by immunofluorescence that human amnion at WG9 consists of TFAP2A+ amniotic ectoderm and PDGFRA+/GATA6+ amniotic mesoderm. To univocally reveal the molecular signature of the extraembryonic mesoderm covering the amniotic ectoderm will require the generation of new single-cell RNA-seq (scRNA-seq) datasets from additional human embryos, including the annotation and further validation of the cell types that form the amnion as well as other extraembryonic structures.

The application of the BMEx overlay primes hPSCs to gain competency to efficiently differentiate to hPGCLCs. Priming hPSCs for 3 h was sufficient, and we report that (some component[s] in) BMEx acted directly and quickly to potentiate BMP signaling via pSMAD1/5/9. The ECM components of BMEx may directly interact with BMP4 such as in *Drosophila*, where BMP4 homolog *Dpp* binds to collagen type IV, mediating BMP signaling.[34] Alternatively, cell-ECM interactions may change the availability and activity of the BMP receptors. For example, ECM-integrin interactions reorder membrane into caveolae-rich lipid raft domains,[35] which is where BMP reeceptor type I (BMPRI) receptors are typically localized, affecting their activity.[36–39] Finally, integrins activate a multitude of downstream pathways that could result in crosstalk with BMP/SMAD signaling.

Downstream of BMP4, BMEx-treated hPSCs showed increased pSMAD1/5/9, leading to upregulation of GATA3, TFAP2A, CDX2, and indirectly of EOMES. EOMES is essential for hPGCLC formation,[6,19] but its continuous and high expression has been shown to promote differentiation to endoderm.[26,27] Consistent with this, EOMES is moderately expressed in day 2 progenitor cells exposed to the BMEx overlay. Moreover, in agreement with EOMES being a direct target of TGF-β/ActA signaling, the addition of ActA increases EOMES expression, resulting in a reduction in hPGCLC yield and a shift toward differentiation to SOX17+/FOXA2+ endoderm-like cells. Surprisingly, in the absence of BMEx, the day 2 progenitors fail to upregulate EOMES. This may explain why 3D differentiation methods have relied on ActA/CHIR99021 pre-induction, which results in *EOMES* expression.

Using the BMEx overlay method, we were able to differentiate hPGCLCs robustly from most hiPSC lines tested, but two lines showed consistently lower differentiation efficiency. A previous analysis of hiPSC lines from 317 individuals showed that hiPSCs display different levels of expression of genes, such as *GATA4*, *GATA6*, *EOMES*, *CER1*, and *NODAL*.[40] While endogenous NODAL signaling is required for hPGCLC differentiation, increased NODAL signaling (by adding ActA) severely hampered hPGCLC differentiation. In agreement, we observed that the two inefficient hiPSC lines F20 and M72 have higher levels of *NODAL* than the efficient lines F99 and M54. Although additional iPSC lines need to be investigated, our observations suggest that hPSCs lines with low levels of endogenous *NODAL* may have higher capacity to undergo hPGCLC differentiation.

The observation that BMEx potentiates BMP/SMAD signaling has significance beyond the field of IVG. BMP4 is widely used in various differentiation protocols and models of early embryogenesis.[12,13,41,42] Moreover, the presence of BMEx has proven to be beneficial for the development of somite-like structures in a mouse 3D gastruloid stem cell model[43] as well as for developing a non-human primate peri-implantation assay.[44] Hence, the combination of BMEx and treatment with BMP4 may prove beneficial to mimic *in vivo* processes more accurately in human models of early embryogenesis.

Limitations of the study

We established that a BMEx overlay method resulted in robust differentiation of hPGCLCs for the majority of the tested hPSC lines, including the commonly used ESC line H1. However, hPSC lines F20 and M72 were characterized by low hPGCLC yields. The mechanism for this line-dependent variability remains unclear, and users of the presented method will have to test hPSC lines for compatibility. In addition, the presented method is reliant on BMEx isolated from murine Engelbreth-Holm-Swarm (EHS) tumor, which is a complex mix of biologically active compounds that may influence differentiation outcome, including trace amounts of growth factors. Hence, the method

presented is neither chemically defined nor clinical grade. For this purpose, the BMEx components that play a role in hPGCLC induction need to be determined.

STAR★METHODS

Detailed methods are provided in the online version of this paper and include the following:

- KEY RESOURCES TABLE
- RESOURCE AVAILABILITY
  - Lead contact
  - Materials availability
  - Data and code availability
- EXPERIMENTAL MODEL AND SUBJECT DETAILS
  - Human samples and ethics statement
  - Routine hPSCs culture
- METHOD DETAILS
  - 2D hPGCLC differentiation
  - CRISPR-Cas9 mediated generation of *POU5F1::EGFP* transgenic hiPSC line
  - Flow cytometry and fluorescence-activated cell sorting (FACS)
  - Reconstitution of fetal ovary and hPGCLCs
  - Immunofluorescence and imaging
  - Wholemount immunofluorescence and imaging
  - Preparation cells for single-cell RNA-sequencing
- QUANTIFICATION AND STATISTICAL ANALYSIS
  - Primary and secondary analysis of single-cell RNA-sequencing data
  - Image quantification and visualization

SUPPLEMENTAL INFORMATION

Supplemental information can be found online at https://doi.org/10.1016/j.crmeth.2023.100488.

ACKNOWLEDGMENTS

We would like to thank the patients who donated the human fetal material used in this study as well as the staff of the Gynaikon Clinic in Rotterdam and het Vrelingshuis in Utrecht. We thank X. Fan for technical assistance; members of the Chuva de Sousa Lopes group, T. den Hamer, and V. Ramovs for fruitful discussions; and M. Bellin for providing the hiPSC line F20. This work was supported by the Dutch Research Council (VICI-2018-91819642 to A.W.O., Y.W.C., C.M.R., and S.M.C.D.E.S.L.), the Nederlandse organisatie voor gezondheidsonderzoek en zorginnovatie (ZonMw) (PSIDER-10250022120001 to T.V.D.H. and S.M.C.D.E.S.L.), and the Novo Nordisk Foundation (reNEW NNF21CC0073729 to A.W.O., S.H., C.F., and S.M.C.D.E.S.L.).

AUTHOR CONTRIBUTIONS

Conceptualization, A.W.O., Y.W.C., and S.M.C.D.E.S.L.; methodology, A.W.O., Y.W.C., and I.M.; investigation, A.W.O., Y.W.C., C.M.R., V.F.V.D.S., S.H., T.V.D.H., I.M., and C.F.; formal analysis (bioinformatics), A.W.O., Y.W.C., C.M.R., V.F.V.D.S., S.H., T.V.D.H., M.A.F.V.G., I.M., C.F., H.M., and S.M.C.D.E.S.L.; writing, A.W.O., Y.W.C., C.M.R., V.F.V.D.S., S.H., T.V.D.H., I.M., M.A.F.V.G., C.F., H.M., and S.M.C.D.E.S.L.; resources, S.H., T.V.D.H., M.A.F.V.G., H.M., C.F., and S.M.C.D.E.S.L.; funding acquisition, S.M.C.D.E.S.L.; supervision, M.A.F.V.G., H.M., C.F., and S.M.C.D.E.S.L. All authors approved the final version of the manuscript.

DECLARATION OF INTERESTS

The authors declare no competing interests.

Received: October 11, 2022
Revised: April 2, 2023
Accepted: May 2, 2023
Published: May 23, 2023

REFERENCES

1. Czukiewska, S.M., and Chuva de Sousa Lopes, S.M. (2022). Fetal germ cell development in humans, a link with infertility. Semin. Cell Dev. Biol. *131*, 58–65. https://doi.org/10.1016/j.semcdb.2022.03.035.
2. Lawson, K.A., Dunn, N.R., Roelen, B.A., Zeinstra, L.M., Davis, A.M., Wright, C.V., Korving, J.P., and Hogan, B.L. (1999). Bmp4 is required for the generation of primordial germ cells in the mouse embryo. Genes Dev. *13*, 424–436. https://doi.org/10.1101/gad.13.4.424.
3. Saitou, M., and Hayashi, K. (2021). Mammalian in vitro gametogenesis. Science *374*, eaaz6830. https://doi.org/10.1126/science.aaz6830.
4. Irie, N., Weinberger, L., Tang, W.W.C., Kobayashi, T., Viukov, S., Manor, Y.S., Dietmann, S., Hanna, J.H., and Surani, M.A. (2015). SOX17 is a critical specifier of human primordial germ cell fate. Cell *160*, 253–268. https://doi.org/10.1016/j.cell.2014.12.013.
5. Sasaki, K., Yokobayashi, S., Nakamura, T., Okamoto, I., Yabuta, Y., Kurimoto, K., Ohta, H., Moritoki, Y., Iwatani, C., Tsuchiya, H., et al. (2015). Robust in vitro induction of human germ cell fate from pluripotent stem cells. Cell Stem Cell *17*, 178–194. https://doi.org/10.1016/j.stem.2015.06.014.
6. Kojima, Y., Sasaki, K., Yokobayashi, S., Sakai, Y., Nakamura, T., Yabuta, Y., Nakaki, F., Nagaoka, S., Woltjen, K., Hotta, A., et al. (2017). Evolutionarily distinctive transcriptional and signaling programs drive human germ cell lineage specification from pluripotent stem cells. Cell Stem Cell *21*, 517–532.e5. https://doi.org/10.1016/j.stem.2017.09.005.
7. Fang, F., Iaquinta, P.J., Xia, N., Liu, L., Diao, L., and Reijo Pera, R.A. (2022). Transcriptional control of human gametogenesis. Hum. Reprod. Update *28*, 313–345. https://doi.org/10.1093/humupd/dmac002.
8. Sasaki, K., Nakamura, T., Okamoto, I., Yabuta, Y., Iwatani, C., Tsuchiya, H., Seita, Y., Nakamura, S., Shiraki, N., Takakuwa, T., et al. (2016). The germ cell fate of cynomolgus monkeys is specified in the nascent amnion. Dev. Cell *39*, 169–185. https://doi.org/10.1016/j.devcel.2016.09.007.
9. Eakin, G.S., and Behringer, R.R. (2004). Gastrulation in other mammals and humans. In Gastrulation: from cells to embryo, C.D. Stern, ed. (Cold Spring Harbour Laboratory Press), pp. 275–287.
10. Chuva de Sousa Lopes, S.M., Roelen, B.A.J., Lawson, K.A., and Zwijsen, A. (2022). The development of the amnion in mice and other amniotes. Philos. Trans. R. Soc. Lond. B Biol. Sci. *377*, 20210258. https://doi.org/10.1098/rstb.2021.0258.
11. Chen, D., Sun, N., Hou, L., Kim, R., Faith, J., Aslanyan, M., Tao, Y., Zheng, Y., Fu, J., Liu, W., et al. (2019). Human primordial germ cells are specified from lineage-primed progenitors. Cell Rep. *29*, 4568–4582.e5. https://doi.org/10.1016/j.celrep.2019.11.083.
12. Zheng, Y., Xue, X., Shao, Y., Wang, S., Esfahani, S.N., Li, Z., Muncie, J.M., Lakins, J.N., Weaver, V.M., Gumucio, D.L., and Fu, J. (2019). Controlled modelling of human epiblast and amnion development using stem cells. Nature *573*, 421–425. https://doi.org/10.1038/s41586-019-1535-2.
13. Zheng, Y., Yan, R.Z., Sun, S., Kobayashi, M., Xiang, L., Yang, R., Goedel, A., Kang, Y., Xue, X., Esfahani, S.N., et al. (2022). Single-cell analysis of embryoids reveals lineage diversification roadmaps of early human development. Cell Stem Cell *29*, 1402–1419.e8. https://doi.org/10.1016/j.stem.2022.08.009.
14. Chang, Y.W., Overeem, A.W., Roelse, C.M., Fan, X., Freund, C., and Chuva de Sousa Lopes, S.M. (2021). Tissue of origin, but not XCI state,

influences germ cell differentiation from human pluripotent stem cells. Cells *10*, 2400. https://doi.org/10.3390/cells10092400.

15. Chen, D., Liu, W., Lukianchikov, A., Hancock, G.V., Zimmerman, J., Lowe, M.G., Kim, R., Galic, Z., Irie, N., Surani, M.A., et al. (2017). Germline competency of human embryonic stem cells depends on eomesodermin. Biol. Reprod. *97*, 850–861. https://doi.org/10.1093/biolre/iox138.

16. Yokobayashi, S., Okita, K., Nakagawa, M., Nakamura, T., Yabuta, Y., Yamamoto, T., and Saitou, M. (2017). Clonal variation of human induced pluripotent stem cells for induction into the germ cell fate. Biol. Reprod. *96*, 1154–1166. https://doi.org/10.1093/biolre/iox038.

17. Taniguchi, K., Shao, Y., Townshend, R.F., Tsai, Y.H., DeLong, C.J., Lopez, S.A., Gayen, S., Freddo, A.M., Chue, D.J., Thomas, D.J., et al. (2015). Lumen Formation is an intrinsic property of isolated human pluripotent stem cells. Stem Cell Rep. *5*, 954–962. https://doi.org/10.1016/j.stemcr.2015.10.015.

18. Karzbrun, E., Khankhel, A.H., Megale, H.C., Glasauer, S.M.K., Wyle, Y., Britton, G., Warmflash, A., Kosik, K.S., Siggia, E.D., Shraiman, B.I., and Streichan, S.J. (2021). Human neural tube morphogenesis in vitro by geometric constraints. Nature *599*, 268–272. https://doi.org/10.1038/s41586-021-04026-9.

19. Kobayashi, T., Zhang, H., Tang, W.W.C., Irie, N., Withey, S., Klisch, D., Sybirna, A., Dietmann, S., Contreras, D.A., Webb, R., et al. (2017). Principles of early human development and germ cell program from conserved model systems. Nature *546*, 416–420. https://doi.org/10.1038/nature22812.

20. Mishra, S., Taelman, J., Chang, Y.W., Boel, A., De Sutter, P., Heindryckx, B., and Chuva De Sousa Lopes, S.M. (2021). Sex-specific isolation and propagation of human premeiotic fetal germ cells and germ cell-like cells. Cells *10*, 1214. https://doi.org/10.3390/cells10051214.

21. Etoc, F., Metzger, J., Ruzo, A., Kirst, C., Yoney, A., Ozair, M.Z., Brivanlou, A.H., and Siggia, E.D. (2016). A balance between secreted inhibitors and edge sensing controls gastruloid self-organization. Dev. Cell *39*, 302–315. https://doi.org/10.1016/j.devcel.2016.09.016.

22. Nemashkalo, A., Ruzo, A., Heemskerk, I., and Warmflash, A. (2017). Morphogen and community effects determine cell fates in response to BMP4 signaling in human embryonic stem cells. Development *144*, 3042–3053. https://doi.org/10.1242/dev.153239.

23. Jo, K., Teague, S., Chen, B., Khan, H.A., Freeburne, E., Li, H., Li, B., Ran, R., Spence, J.R., and Heemskerk, I. (2022). Efficient differentiation of human primordial germ cells through geometric control reveals a key role for Nodal signaling. Elife *11*, e72811. https://doi.org/10.7554/eLife.72811.

24. Tyser, R.C.V., Mahammadov, E., Nakanoh, S., Vallier, L., Scialdone, A., and Srinivas, S. (2021). Single-cell transcriptomic characterization of a gastrulating human embryo. Nature *600*, 285–289. https://doi.org/10.1038/s41586-021-04158-y.

25. Li, L., Dong, J., Yan, L., Yong, J., Liu, X., Hu, Y., Fan, X., Wu, X., Guo, H., Wang, X., et al. (2017). Single-cell RNA-seq analysis maps development of human germline cells and gonadal niche interactions. Cell Stem Cell *20*, 858–873.e4. https://doi.org/10.1016/j.stem.2017.03.007.

26. Heslop, J.A., Pournasr, B., and Duncan, S.A. (2022). Chromatin remodeling is restricted by transient GATA6 binding during iPSC differentiation to definitive endoderm. iScience *25*, 104300. https://doi.org/10.1016/j.isci.2022.104300.

27. Yoney, A., Bai, L., Brivanlou, A.H., and Siggia, E.D. (2022). Mechanisms underlying WNT-mediated priming of human embryonic stem cells. Development *149*, dev200335. https://doi.org/10.1242/dev.200335.

28. Hwang, Y.S., Suzuki, S., Seita, Y., Ito, J., Sakata, Y., Aso, H., Sato, K., Hermann, B.P., and Sasaki, K. (2020). Reconstitution of prospermatogonial specification in vitro from human induced pluripotent stem cells. Nat. Commun. *11*, 5656. https://doi.org/10.1038/s41467-020-19350-3.

29. Yamashiro, C., Sasaki, K., Yabuta, Y., Kojima, Y., Nakamura, T., Okamoto, I., Yokobayashi, S., Murase, Y., Ishikura, Y., Shirane, K., et al. (2018). Generation of human oogonia from induced pluripotent stem cells in vitro. Science *362*, 356–360. https://doi.org/10.1126/science.aat1674.

30. Chen, X., Janssen, J.M., Liu, J., Maggio, I., t Jong, A.E.J., Mikkers, H.M.M., and Gonçalves, M.A.F.V. (2017). In trans paired nicking triggers seamless genome editing without double-stranded DNA cutting. Nat. Commun. *8*, 657. https://doi.org/10.1038/s41467-017-00687-1.

31. Chen, X., Tasca, F., Wang, Q., Liu, J., Janssen, J.M., Brescia, M.D., Bellin, M., Szuhai, K., Kenrick, J., Frock, R.L., and Gonçalves, M.A.F.V. (2020). Expanding the editable genome and CRISPR-Cas9 versatility using DNA cutting-free gene targeting based on in trans paired nicking. Nucleic Acids Res. *48*, 974–995. https://doi.org/10.1093/nar/gkz1121.

32. Wang, Q., Liu, J., Janssen, J.M., Le Bouteiller, M., Frock, R.L., and Gonçalves, M.A.F.V. (2021). Precise and broad scope genome editing based on high-specificity Cas9 nickases. Nucleic Acids Res. *49*, 1173–1198. https://doi.org/10.1093/nar/gkaa1236.

33. Sebastiano, V., Kang, G., Vijayakumar, S., Sala, R., Chen, A., Adebayo, A., Cipriano, A., Fowler, J., Ang, L.T., and Loh, K. (2021). Monolayer platform to generate and purify human primordial germ cells in vitro provides new insights into germline specification. Preprint at Research Square. https://doi.org/10.21203/rs.3.rs-113078/v1.

34. Wang, X., Harris, R.E., Bayston, L.J., and Ashe, H.L. (2008). Type IV collagens regulate BMP signalling in Drosophila. Nature *455*, 72–77. https://doi.org/10.1038/nature07214.

35. Kim, S.H., Turnbull, J., and Guimond, S. (2011). Extracellular matrix and cell signalling: the dynamic cooperation of integrin, proteoglycan and growth factor receptor. J. Endocrinol. *209*, 139–151. https://doi.org/10.1530/JOE-10-0377.

36. Bonor, J., Adams, E.L., Bragdon, B., Moseychuk, O., Czymmek, K.J., and Nohe, A. (2012). Initiation of BMP2 signaling in domains on the plasma membrane. J. Cell. Physiol. *227*, 2880–2888. https://doi.org/10.1002/jcp.23032.

37. Ehrlich, M. (2016). Endocytosis and trafficking of BMP receptors: regulatory mechanisms for fine-tuning the signaling response in different cellular contexts. Cytokine Growth Factor Rev. *27*, 35–42. https://doi.org/10.1016/j.cytogfr.2015.12.008.

38. Hartung, A., Bitton-Worms, K., Rechtman, M.M., Wenzel, V., Boergermann, J.H., Hassel, S., Henis, Y.I., and Knaus, P. (2006). Different routes of bone morphogenic protein (BMP) receptor endocytosis influence BMP signaling. Mol. Cell Biol. *26*, 7791–7805. https://doi.org/10.1128/MCB.00022-06.

39. Ramos, M., Lamé, M.W., Segall, H.J., and Wilson, D.W. (2006). The BMP type II receptor is located in lipid rafts, including caveolae, of pulmonary endothelium in vivo and in vitro. Vascul. Pharmacol. *44*, 50–59. https://doi.org/10.1016/j.vph.2005.09.007.

40. Carcamo-Orive, I., Hoffman, G.E., Cundiff, P., Beckmann, N.D., D'Souza, S.L., Knowles, J.W., Patel, A., Papatsenko, D., Abbasi, F., Reaven, G.M., et al. (2017). Analysis of transcriptional variability in a large human iPSC library reveals genetic and non-genetic determinants of heterogeneity. Cell Stem Cell *20*, 518–532.e9. https://doi.org/10.1016/j.stem.2016.11.005.

41. Simunovic, M., Metzger, J.J., Etoc, F., Yoney, A., Ruzo, A., Martyn, I., Croft, G., You, D.S., Brivanlou, A.H., and Siggia, E.D. (2019). A 3D model of a human epiblast reveals BMP4-driven symmetry breaking. Nat. Cell Biol. *21*, 900–910. https://doi.org/10.1038/s41556-019-0349-7.

42. Warmflash, A., Sorre, B., Etoc, F., Siggia, E.D., and Brivanlou, A.H. (2014). A method to recapitulate early embryonic spatial patterning in human embryonic stem cells. Nat. Methods *11*, 847–854. https://doi.org/10.1038/nmeth.3016.

43. van den Brink, S.C., Alemany, A., van Batenburg, V., Moris, N., Blotenburg, M., Vivié, J., Baillie-Johnson, P., Nichols, J., Sonnen, K.F., Martinez Arias, A., and van Oudenaarden, A. (2020). Single-cell and spatial transcriptomics reveal somitogenesis in gastruloids. Nature *582*, 405–409. https://doi.org/10.1038/s41586-020-2024-3.

44. Yang, R., Goedel, A., Kang, Y., Si, C., Chu, C., Zheng, Y., Chen, Z., Gruber, P.J., Xiao, Y., Zhou, C., et al. (2021). Amnion signals are essential for mesoderm formation in primates. Nat. Commun. *12*, 5126. https://doi.org/10.1038/s41467-021-25186-2.

45. Hao, Y., Hao, S., Andersen-Nissen, E., Mauck, W.M., 3rd, Zheng, S., Butler, A., Lee, M.J., Wilk, A.J., Darby, C., Zager, M., et al. (2021). Integrated analysis of multimodal single-cell data. Cell *184*, 3573–3587.e29. https://doi.org/10.1016/j.cell.2021.04.048.

46. Schindelin, J., Arganda-Carreras, I., Frise, E., Kaynig, V., Longair, M., Pietzsch, T., Preibisch, S., Rueden, C., Saalfeld, S., Schmid, B., et al. (2012). Fiji: an open-source platform for biological-image analysis. Nat. Methods *9*, 676–682. https://doi.org/10.1038/nmeth.2019.

47. Wickham, H., Averick, M., Bryan, J., Chang, W., D'Agostino McGowan, L., François, R., Grolemund, G., Hayes, A., Henry, L., Hester, J., et al. (2019). Welcome to the tidyverse. J. Open Source Softw. *4*, 1686. https://doi.org/10.21105/joss.01686.

48. Blighe, K., Rana, S., and Lewis, M. EnhancedVolcano: Publication-ready volcano plots with enhanced colouring and labeling. R package version 1.18.0. https://github.com/kevinblighe/EnhancedVolcano.

49. Kolde, R. (2019). pheatmap: Pretty Heatmaps. R package version 1.0.12. https://cran.r-project.org/web/packages/pheatmap/index.html.

50. Wickham, H. (2016). Ggplot2: Elegant Graphics for Data Analysis (Springer-Verlag).

51. Gu, Z., Eils, R., and Schlesner, M. (2016). Complex heatmaps reveal patterns and correlations in multidimensional genomic data. Bioinformatics *32*, 2847–2849. https://doi.org/10.1093/bioinformatics/btw313.

52. van den Brink, S.C., Sage, F., Vértesy, Á., Spanjaard, B., Peterson-Maduro, J., Baron, C.S., Robin, C., and van Oudenaarden, A. (2017). Single-cell sequencing reveals dissociation-induced gene expression in tissue subpopulations. Nat. Methods *14*, 935–936. https://doi.org/10.1038/nmeth.4437.

STAR★METHODS

KEY RESOURCES TABLE

REAGENT or RESOURCE	SOURCE	IDENTIFIER
**Antibodies**		
Mouse anti-OCT3/4 (POU5F1) (1:200)	Santa Cruz Biotechnology	Cat# sc-5279; RRID: AB_628051
Goat anti-SOX17 (1:500)	R&D Systems	Cat# AF1924; RRID: AB_355060
Rat anti-CD49f (1:200)	Thermo Fisher Scientific	Cat# 14-0495-82; RRID: AB_891480
BV421 anti-CD49f (ITGA6) (1:200)	Biolegend	Cat# 313623; RRID: AB_2562243
PEcy7 anti-CD325 (EPCAM) (1:200)	Biolegend	Cat# 324222; RRID: AB_2561506
Goat anti-Alkaline Phosphatase (ALPL) (1:500)	R&D Systems	Cat# AF2910; RRID: AB_664062
Mouse anti-Podoplanin (PDPN) (1:200)	Abcam	Cat# ab256561; RRID: N/A
Rat anti-Blimp1 (PRDM1) (1:200)	Invitrogen	Cat# 14-5963-82; RRID: AB_1907437
Rabbit anti-AP2γ (TFAP2C) (1:200)	Cell Signaling Technology	Cat# 2320; RRID: AB_2202287
Mouse anti-AP2a (TFAP2A) (1:200)	Santa Cruz Biotechnology	Cat# sc-12726; RRID: AB_667767
Goat anti-GATA6 (1:500)	R&D Systems	Cat# AF1700; RRID: AB_2108901
Rabbit anti-PDGFRa (1:500)	Cell Signaling Technology	Cat# 5241; RRID: AB_10692773
Goat anti-HAND1 (1:200)	R&D Systems	Cat# AF3168; RRID: AB_2115853
Mouse anti-GATA3 (1:200)	Thermo Fisher Scientific	Cat# MA1-028; RRID: AB_2536713
Mouse anti-Cytokeratin 7 (KRT7) (1:200)	Thermo Fisher Scientific	Cat# MA1-06316; RRID: AB_559789
Rabbit anti-Slug (SNAI2) (1:200)	Cell Signaling	Cat# 9585; RRID: AB_2239535
Mouse anti-Integrin β1 (ITGB1) (1:200)	Santa Cruz Biotechnology	Cat# sc-53711; RRID: AB_629021
Rabbit anti-Laminin (panLAM) (1:100)	Abcam	Cat# ab11575; RRID: AB_298179
Mouse anti-β-catenin (CTNNB1) (1:500)	BD Biosciences	Cat# 610154; RRID: AB_397555
Rabbit anti-Podocalyxin (PODXL) (1:500)	R&D Systems	Cat# AF1658; RRID: AB_354920
Rabbit anti-ZO1 (TJP1) (1:500)	Thermo Fisher Scientific	Cat# 61–7300; RRID: AB_2533938
Rabbit anti-Phospho-SMAD1 (Ser463/465)/ SMAD5 (Ser463/465)/SMAD9 (Ser465/467) (1:200)	Cell Signaling Technology	Cat# 13820; RRID: AB_2493181
Mouse anti-CDX2 (1:200)	Biogenex	Cat# MU392-UC; RRID: AB_2335627
Goat anti-SOX2 (1:200)	Santa Cruz Biotechnology	Cat# sc-17319; RRID: AB_661259
Rabbit anti-EOMES (1:200)	Abcam	Cat# ab23345; RRID: AB_778267
Rabbit anti-FOXA2 (1:200)	Merck Millipore	Cat# 07–633; RRID: AB_390153
Rabbit Anti-DDX4/VASA (1:500)	Abcam	ab13840; RRID:AB_443012
Goat anti-DDX4/VASA (1:500)	R&D Systems	AF2030; RRID:AB_2277369
Rabbit Anti-DAZL (1:500)	Abcam	ab215718; RRID:AB_2893177
Chicken Anti-GFP (1:600)	Abcam	ab13970; RRID:AB_300798
Alexa Flour 488 donkey anti-mouse IgG (1:500)	Thermo Fisher Scientific	Cat# A-21202; RRID: AB_141607
Alexa Flour 555 donkey anti-rabbit IgG (1:500)	Thermo Fisher Scientific	Cat# A-31572; RRID: AB_162543
Alexa Flour 647 donkey anti-goat IgG (1:500)	Thermo Fisher Scientific	Cat# A-21447; RRID: AB_2535864
Alexa Fluor 555 donkey anti-chicken IgY (1:500)	Thermo Fisher Scientific	Cat# A78949; RRID:AB_2921071
Alexa Flour 555 donkey anti-rat IgG (1:500)	Thermo Fisher Scientific	Cat# A48270; RRID: AB_2896336
**Biological samples**		
Human fetal amnion; age in weeks of gestation: WG9; sex: male	Abortion clinic Gynaikon, Rotterdam, the Netherlands	N/A
Human fetal ovary; age in weeks of gestation: WG19; sex: female	Abortion clinic het Vrelingshuis, Utrecht, the Netherlands	N/A

(*Continued on next page*)

Continued

REAGENT or RESOURCE	SOURCE	IDENTIFIER
Chemicals, peptides, and recombinant proteins		
7-AAD Viability Staining Solution	Biolegend	Cat# 420403
Accutase	Stem Cell Technologies	Cat# 07920
Advanced RPMI 1640 Medium	Thermo Fisher Scientific	Cat# 12633012
B-27 Supplement	Thermo Fisher Scientific	Cat# 17504-044
Bovine Serum Albumin (BSA)Fraction V	Sigma Aldrich	Cat# 10735086001
Cultrex Stem Cell Qualified, Reduced Growth Factor Basement Membrane Extract	R&D Systems	Cat# 3434-010-02<br>Lot#: 1659223 (A), 1677279 (B)
Geltrex LDEV-Free, hESC-Qualified, Reduced Growth Factor Basement Membrane Matrix	Thermo Fisher Scientific	Cat# A1413302<br>Lot#: 963718 (A), 963726 (B), 2327546 (C)
Matrigel hESC-Qualified Matrix, LDEV-free	Corning	Cat# 354277<br>Lot#: 1341001 (A), 1235001 (B)
DAPI (4′,6-Diamidino-2-Phenylindole)	Thermo Fisher Scientific	Cat# D3571
DMEM/F-12, GlutaMAX	Thermo Fisher Scientific	Cat# 10565018
DPBS, no calcium, no magnesium	Thermo Fisher Scientific	Cat# 14190144
GlutaMAX Supplement	Thermo Fisher Scientific	Cat# 35050061
MEM Non-Essential Amino Acids Solution	Thermo Fisher Scientific	Cat# 11140050
mTeSR-Plus	Stem Cell Technologies	Cat# 100-0276
MycoZap Plus-CL	Lonza	Cat# VZA-2011
NaCl 0.9%	Fresenius Kabi	Cat# 14557487
Paraformaldehyde (PFA)	Sigma Aldrich	Cat# 1040051000
ProLong Gold Antifade Mountant	Thermo Fisher Scientific	Cat# P36930
Recombinant Human BMP4	R&D Systems	Cat# 314-BP-050
Recombinant Human EGF	R&D Systems	Cat# 236-EG-200
Recombinant Human LIF	Peprotech	Cat# 300-05
Recombinant Human SCF	R&D Systems	Cat# 11010-SC-100
Recombinant Human/Mouse/Rat Activin A	R&D Systems	Cat# 338-AC-050/CF
ReLeSR	Stem Cell Technologies	Cat# 05872
Revitacell	Thermo Fisher Scientific	Cat# A2644501
SB431542	Tocris	Cat# 1614/10
Triton X-100	Sigma Aldrich	Cat# T8787
TrypLE Express Enzyme	Thermo Fisher Scientific	Cat# 12604013
TWEEN 20	Sigma Aldrich	Cat# 8.22184
UltraPure 0.5M EDTA	Thermo Fisher Scientific	Cat# 15575020
Y-27632 (ROCKi)	Tocris	Cat# 72302
Forskolin	Biogems	Cat# 6652995
L-Ascorbic acid	Sigma Aldrich	Cat# A92902
Agarose, Low Melting Point, Analytical Grade	Promega	Cat# V2111
TAT-CRE Recombinase	Sigma Aldrich	Cat# SCR508
Lipofectamine Stem Transfection Reagent	Thermo Fisher Scientific	Cat# STEM00003
Puromycin	Invivogen	Cat# ant-pr-1
Opti-MEM I Reduced Serum Medium	Thermo Fisher Scientific	Cat# 31985062
Donkey Serum	Sigma Aldrich	Cat# D9663
Pierce BCA Protein Assay kit	Thermo Fisher Scientific	Cat# 23225
Critical commercial assays		
Chromium Next GEM Single Cell 3′ HT Kit v3.1	10x Genomics	Cat# PN-1000348
Chromium Next GEM Chip M Single Cell Kit	10x Genomics	Cat# PN-1000349
Dual Index Kit TT Set A	10x Genomics	Cat# PN-1000215

(*Continued on next page*)

Continued		
REAGENT or RESOURCE	SOURCE	IDENTIFIER
Deposited data		
Raw and processed scRNA-seq data	This work	GSE214521
Single-cell RNA-seq from hPSC line UCLA2 during hPGCLC differentiation	Chen et al.[11]	GSE140021
Single-cell RNA-seq from hPSC lines used for amniotic sac embryoids/µPASE model	Zheng et al.[13]	GSE185643
Single-cell RNA-seq from a CS7 human embryo	Tyser et al.[24]	E-MTAB-9388
Single-cell RNA-seq from human fetal gonads	Li et al.[25]	GSE86146
Experimental models: Cell lines		
H1 hESC line (WA01) (male)	WiCell	NIH registration no. 0043; hPSCreg: WAe001-A; https://hpscreg.eu/cell-line/Wae001-A
M54 hiPSC line (male, kidney epithelial cells/urine, sendai)	LUMC iPSC core facility	LUMC0054iCTRL03; hPSCreg: LUMCi001-B; https://hpscreg.eu/cell-line/LUMCi001-B
F99 hiPSC line (female, skin, RNA), same donor as F31	LUMC iPSC core facility	LUMC0099iCTRL04; hPSCreg: LUMCi004-A; https://hpscreg.eu/cell-line/LUMCi004-A
F31 hiPSC line (female, kidney epithelial cells/urine, episomal), same donor as F99	LUMC iPSC core facility	LUMC0031iCTRL08; hPSCreg: LUMCi004-C; https://hpscreg.eu/cell-line/LUMCi004-C
F20 hiPSC line (female, skin, sendai)	LUMC iPSC core facility	LUMC0020iCTRL06; hPSCreg: LUMCi028-A; https://hpscreg.eu/cell-line/LUMCi028-A
M72 hiPSC line (male, skin, RNA)	LUMC iPSC core facility	LUMC0072iCTRL01; hPSCreg: LUMCi029-A, https://hpscreg.eu/cell-line/LUMCi029-A
F198 hiPSC line (female, kidney epithelial cells/urine, RNA)	LUMC iPSC core facility	N/A
F198 hiPSC line with POU5F1:GFP	This study	N/A
Recombinant DNA		
AX74_pDonorOCT4.TS	Chen et al.[31]	
AX33_pgRNAOCT4.1	Chen et al.[31]	
AB65_pCAG.Cas9D10A.rBGpA	Chen et al.[31]	
Software and algorithms		
R v4.1.2 and v4.0.5	R Core Team (2020)	https://www.r-project.org
Rstudio	Rstudio Team (2020)	http://www.rstudio.com/
Seurat v4.0.5 and v4.1.1	Hao et al.[45]	https://satijalab.org/seurat/index.html
Fiji (ImageJ)	Schindelin et al.[46]	https://imagej.net/software/fiji/
Cell Ranger v6.1.1	10X Genomics	https://support.10xgenomics.com/single-cell-gene-expression/software/overview/welcome
Flowjo v10.8.1	Flowjo	https://www.flowjo.com/
ggplot2 v 3.3.5	Wickham[47]	https://ggplot2.tidyverse.org/
Enhanced Volcano v1.12.0	Blighe et al.[48]	https://rdrr.io/bioc/EnhancedVolcano/man/EnhancedVolcano.html
Pheatmap v1.0.12	Kolde[49]	https://github.com/raivokolde/pheatmap
Tidyverse v1.3.1	Wickham et al.[50]	https://tidyverse.tidyverse.org/
ComplexHeatmap v2.14.0	Gu et al.[51,48]	http://bioconductor.org/packages/devel/bioc/html/ComplexHeatmap.html
Adobe Photoshop v 22.1.1	Adobe	https://www.adobe.com
Imaris (Oxford Instruments)	Imaris	https://imaris.oxinst.com/
Custom code repository	This work	https://doi.org/10.5281/zenodo.7875002
Other		
Round coverslip glasses, Menzel Gläser, 10mm	VWR	Cat# 630-2115
Corning 40µm Cell Strainer	Corning	Cat# 431750
µ-Slide 18 Well	Ibidi	Cat# 81816

(*Continued on next page*)

Continued

REAGENT or RESOURCE	SOURCE	IDENTIFIER
Starfrost microscope slides	Knittel	Cat# 3056-1
Falcon 5 mL Round Bottom Polystyrene Test Tube, with Cell Strainer Snap Cap	Corning	Cat# 352235
PrimeSurface 96 wells, low attachment,V bottom	S-Bio	Cat# MS-9096VZ

RESOURCE AVAILABILITY

Lead contact

Further information and requests for resources and reagents should be directed to and will be fulfilled by the lead contact Susana M. Chuva de Sousa Lopes (lopes@lumc.nl).

Materials availability

This study did not generate new unique materials or reagents.

Data and code availability

- Single-cell RNA-seq data have been deposited at GEO and are publicly available as of the date of publication. Accession numbers are listed in the key resources table.
- The code used here is available at https://github.com/johnmous/single_cellhPGCLCs. All original code has also been deposited at Zenodo and is publicly available as of the date of publication. DOI is listed in the key resources table.
- Any additional information required to reanalyze the data reported in this paper is available from the lead contact upon request.

EXPERIMENTAL MODEL AND SUBJECT DETAILS

Human samples and ethics statement

All experiments performed in this study were carried out strictly under the guidelines specified in the Declaration of Helsinki for Medical Research involving Human Subjects. For ethics approval, a letter of no objection was issued by the Medical Ethical Committee of Leiden University Medical Center (B21.054).

The human amnion and fetal ovary samples used were collected from elective abortions without medical indication, after obtaining informed consent from the donors. The amnion (2 cm × 2 cm fragment) was dissected in 0.9% NaCl solution (Fresenius Kabi), fixed in 4% paraformaldehyde (PFA) (Sigma) overnight (o/n) at 4°C washed three time in PBS, and transferred to 70% ethanol for storage at 4°C until further use.

The fetal ovary was dissected into small pieces and cultured o/n in aRB27 basal medium [advanced RPMI1640 (Thermo Fisher Scientific) supplemented with B27 (1:100) (Thermo Fisher Scientific), 1× Glutamax (Thermo Fisher Scientific), 1× MEM Non-Essential Amino Acids (Thermo Fisher Scientific) and Mycozap (Lonza)] plus RevitaCell supplement (Thermo Fisher Scientific), before being used next day for reconstitution.

Routine hPSCs culture

The hPSCs used in this study were either purchased from WiCell (H1) or obtained from the LUMC hiPSC core facility [LUMC0054iCTRL03 (M54), LUMC0072iCTRL01 (M72), LUMC0020iCTRL06 (F20), LUMC0031iCTRL08 (F31), LUMC0099iCTRL04 (F99), LUMC0198iCTRL01 (F198)]. All hPSC lines were cultured in mTeSR-Plus media (STEMCELL Technologies) supplemented with MycoZap (Lonza), to prevent bacterial, fungal and mycoplasma contamination, on tissue culture plates coated with either Geltrex (Thermo Fisher Scientific) or Cultrex (R&D Systems) diluted in DMEM/F12 (Thermo Fisher Scientific) at 1% (v/v) concentration. Cells were cultured at 37°C in a humidified normoxic incubator with 5% CO_2. Routine clump passaging was performed every 4–7 days using ReLeSR (Stem Cell Technologies). The starting cultures were karyotypically normal and were used for no more than 20 passages.

METHOD DETAILS

2D hPGCLC differentiation

High quality hiPSCs of 60–80% confluency with minimal differentiation were used for hPGCLC differentiation. Briefly, cells were dissociated with TryPLE (Thermo Fisher Scientific) at 37°C for 5 min (min), diluted in DMEM/F12 (Thermo Fisher Scientific) to stop digestion, and spun down. Single cells were resuspended in cold mTeSR-Plus media containing RevitaCell supplement (Thermo Fisher Scientific) and 2% Geltrex (LDEV-free, hESC-Qualified, Reduced Growth Factor) or Cultrex (Stem Cell Qualified Reduced

Growth Factor) at 2.04 × 10^5 cells/mL. We have obtained comparable hPGCLC differentiation using 10μM Y-27632 (Stem Cell Technologies) instead of RevitaCell. Depending on the plate format, the desired volume of cell suspension was added to the Geltrex- or Cultrex-coated plate to achieve a final plating density of 60,000 cells/cm^2.

On Day 1 (24 h after plating), the medium was aspirated and the cells were washed once with aRB27 basal medium [advanced RPMI1640 (Thermo Fisher Scientific) supplemented with B27 (1:100) (Thermo Fisher Scientific), 1× Glutamax (Thermo Fisher Scientific), 1× MEM Non-Essential Amino Acids (Thermo Fisher Scientific) and Mycozap (Lonza)]. After washing, the differentiation media consisting of aRB27 with 2% Geltrex or Cultrex (+BMEx overlay) and 10 ng/mL BMP4 (R&D Systems) was added to the cells [or with variations: omission of BMEx, varying BMP4 concentration and addition of SCF, LIF EGF, ActA (R&D Systems) or SB431542 (Tocris)]. Media exchange was performed the next day (D2) and on day 3 (D3), the medium was switched to aRB27 basal medium with 10 ng/mL BMP4, 10 ng/mL human LIF (PeproTech), 50 ng/mL SCF (R&D Systems) and 50 ng/mL EGF (R&D Systems). Medium change was performed daily until D5.

To compare Geltrex, Cultrex and Matrigel (LDEV-free, hESC-Qualified), both the culture surface coating, as well as BMEx supplementation steps were similar. The protein concentration in Cultrex, Geltrex and Matrigel was measured using Pierce BCA Protein Assay kit (Thermo Fisher Scientific) following manufactures instructions using a Glowmax Explorer plate reader (GM3500, Promega).

CRISPR-Cas9 mediated generation of *POU5F1::EGFP* transgenic hiPSC line

The generation of a hiPSC line harboring an *EGFP* knock-in at *POU5F1* was performed by in trans paired nicking genome editing[30] using target site-modified donor construct AX74_pDonorOCT4.TS together with gRNA and nickase plasmids AX33_pgRNAOCT4.1 and AB65_pCAG.Cas9.D10A,rBGpA, respectively, as previously described.[31] Cells of the hiPSC line F198 were dissociated with TryPLE and plated at 50,000 cells/well in wells of a Geltrex-coated 24-well plate in mTeSR-Plus medium containing RevitaCell supplement. After 24 h, the cells were transfected with a mixture of the aforementioned plasmids (200 ng per plasmid), using Lipofectamine Stem Transfection Reagent (Thermo Fisher) according to manufacturer's instructions. After 48 h of incubation period, the cells were treated with 0.5 μg/mL puromycin (Invivogen) for three days. Puromycin-resistant cells were expanded, passaged using TryPLE at 100,000 cells/well in a Geltrex-coated 12-well plate, in mTeSR-Plus medium containing RevitaCell supplement. After 24 h, the cells were incubated overnight with 1 μM TAT-CRE Recombinase in mTeSR-Plus medium. EGFP positive cells were sorted as described below.

Flow cytometry and fluorescence-activated cell sorting (FACS)

Single cell suspension of the hPGCLC differentiation culture was generated by incubating the cells with Accutase (Stem Cell Technologies) for 15 min at 37°C, followed by vigorous pipetting to break up clumps. The cell suspension was then filtered through a 40 μm nylon mesh strainer (Corning), pelleted by centrifugation, washed once in FACS buffer [DPBS (Thermo Fisher Scientific) with 0.5% BSA (Sigma-Aldrich)], pelleted by centrifugation and incubated with conjugated-antibodies diluted in FACS buffer at approximately 0.5-2×10^6 cells/mL at 4°C for 30 min. Thereafter, the cell suspension was pelleted by centrifugation and recovered cells were resuspended in FACS buffer containing 7AAD (BioLegend, 1:100). The flow cytometry analysis was performed on an LSR-II flow cytometer (BD Biosciences) or an LSRFortessa flow cytometer (BD Biosciences). The FACS data were collected from FACSDiva Software (BD Biosciences). The cell sorting was performed on a CytoFLEX SRT benchtop cell sorter (Beckman) and FlowJo Software (BD Biosciences) were used for analysis.

Reconstitution of fetal ovary and hPGCLCs

Human fetal ovary pieces were digested with an enzyme mixture containing 1 mg/mL Collagenase IV (Invitrogen), 0.5 mg/mL Hyaluronidase (Sigma-Aldrich) and 20 U/mL DNAse I (Sigma-Aldrich) for 30–45 min at 37°C, followed by vigorous pipetting to break up cell clumps. The single cell suspension was passed through a 40 μm nylon mesh strainer (Corning). To form the aggregates, 30,000 fetal ovary cells were combined with 5,000 sorted hPGCLCs per well of an ultra-low 96-well V-bottom plate, in aRB27 medium supplemented with RevitaCell. 3 days later, the aggregates were embedded in 1.5% low-melting agarose droplets in aRB27 medium supplemented with 100 ng/mL SCF, 5 μM forskolin (Biogems) and 150 μM ascorbic acid (Sigma-Aldrich). Medium changes were performed every 3 days and on day 17 the medium was switched back to aRB27 basal medium. On day 25, the reconstituted aggregates were isolated by breaking up the agarose droplets and fixed with 4% PFA at 4°C o/n for immunofluorescence analysis.

Immunofluorescence and imaging

Cells cultured for imaging were either cultured on glass coverslips (VWR) or 18-well μ-slides (Ibidi). At the time of analysis, cells were fixed in 4% PFA for 15 min at room temperature (rt). The fixed cells were then washed three times with PBS and permeabilized with 0.3% Triton X-100 (Sigma-Aldrich) diluted in PBS for 15 min at rt, followed by three washes with PBST (0.05% Tween 20 (Sigma-Aldrich) in PBS). Subsequently, cells were treated with blocking buffer (1% BSA (Sigma-Aldrich) diluted in PBST) for 1 h at rt and incubated with primary antibodies diluted in blocking buffer at 4°C o/n. Next, the cells were washed three times with PBST and incubated with secondary antibodies and DAPI (Life Technologies) diluted in blocking buffer for 1 h at rt, followed by three PBS washes. Cells in 18-well μ-slides were imaged directly, whilst cells on glass coverslips were mounted with ProLong Gold (Thermo Fisher Scientific).

For paraffin embedding and imaging of the fetal ovary and hPGCLC/fetal ovary aggregates, samples were fixed in 4% PFA at 4°C o/n. Samples were then embedded in paraffin with a Shandon Excelsior tissue processor (Thermo Fisher Scientific) and subsequently sectioned (5 μm), using an RM2065 microtome (Leica Instruments) and mounted to StarFrost glass slides (Knittel). The sections were

deparaffinized using xylene, and rehydrated with an ethanol dilution ending in water. Antigen retrieval was performed using Tris-EDTA buffer (10 mM Tris, 1 mM EDTA, 0.05% Tween 20, pH 9) for 12 min at 98°C using a TissueWave 2 Microwave (Thermo Fisher Scientific). Slides were rinsed with PBS, and treated with blocking solution (1% BSA and 10% normal donkey serum (Sigma-Aldrich) in PBST) for 1 h at rt. Slides were then incubated with primary antibodies diluted in blocking solution o/n at 37°C, washed three times with PBS, followed by incubation with secondary antibodies and DAPI diluted in blocking solution for 1 h at rt. Finally, samples were washed three times with PBS, covered in ProLong Gold (Thermo Fisher Scientific) and mounted with coverslips.

Imaging was performed on either a 200 Series Dragonfly spinning disk confocal microscope (Andor) or a TCS SP8 confocal microscope (Leica). Renderings of image stacks were generated using the Imaris surface creation functionality. Images were processed in Fiji (ImageJ2) and Adobe Photoshop (Adobe).

Wholemount immunofluorescence and imaging

Amnion (2 cm × 2 cm fragment) was washed three times 30 min in PBS at rt to remove the 70% ethanol solution. The tissue was permeabilized with 0.5% Triton X-100 in PBS for 1 h at rt, followed by o/n blocking in 0.2% Triton X-100 with 1% BSA in PBS (whole mount blocking buffer) at 4°C. The tissue was then incubated with primary antibodies diluted in whole mount blocking buffer for 24 h at 4°C, washed three times in PBST for 15 min at rt and incubated with secondary antibodies diluted in whole mount blocking buffer for 3 h at rt. Finally, the tissue was washed three times with PBS for 15 min, mounted between cover glass and glass slide using Prolong Gold and imaged on a 200 Series Dragonfly spinning disk confocal microscope (Andor).

Preparation cells for single-cell RNA-sequencing

BMEx-overlay cultures (D2 and D5) and hPSCs (D0) were digested with Accutase at 37°C for 15 min (D2 and D5 samples) and 5 min (D0 samples). Samples were washed once in FACS buffer and centrifuged. Cells were then resuspended in FACS buffer, filtered through the FACS tube strainer cap and treated with 7AAD diluted in FACS buffer at 1:100 dilution, on ice for 3 min. Live cells were sorted on a CytoFLEX SRT benchtop cell sorter (Beckman). The collected live cells were sent to the Leiden Genome Technology Center (LGTC) for library preparation using the Chromium Next GEM Single Cell 3′ HT Kit v3.1 (10x Genomics) according to the manufacturers' instructions and sequenced on a NovaSeq6000 with V1.5 chemistry (illumina) at Genome Scan.

QUANTIFICATION AND STATISTICAL ANALYSIS

Primary and secondary analysis of single-cell RNA-sequencing data

Raw RNA sequencing data was processed using the Cell Ranger pipeline (v6.1.1) in which reads were aligned to the human reference genome (GRCh38) and gene UMI count matrices were generated based on gene annotation in Cell Ranger reference annotation version GRCh38-2020-A. The R package Vireo2 (v0.2.3) was used to distinguish cells from different cell lines (N = 4) based on genetic variation. Count matrices generated with Cell Ranger were analyzed using Seurat (v4.1.1) workflow in R (v4.0.5). Functions mentioned below are part of the Seurat workflow unless specified otherwise. For quality control, cells expressing <2000 or >7000 genes or cells having >100000 UMIs were excluded from further analysis. In addition, cells with >10%, or <0.1% of the total UMIs coming from mitochondrial genes were excluded. Cells with >6% of UMIs mapping to dissociation-induced genes were excluded as well.[52] Data was log-normalized using the NormalizeData function (scale factor: 100000). To focus on cell type specific characteristics, batch effect between cell lines (N = 4) was corrected using the fastMNN function from R package batchelor (v1.6.0). The top 2000 variable features (genes) were selected (function: FindVariableFeatures) to perform Principal Component Analysis (PCA) (function: RunPCA). The first 15 principal components (PCs) were used to calculate cell clusters, with resolution parameter set to 0.22 (functions: FindNeighbors, FindClusters). Cells were visualized on a two-dimensional plot calculated using the Uniform Manifold Approximation and Projection (UMAP) algorithm (function: RunUMAP). Differentially expressed genes (DEGs) for each cluster were calculated using function FindAllMarkers (parameters: only.pos = TRUE, min.pct >0.25 and logfc.threshold >0.25). Expression of individual genes were visualized using functions FeaturePlot or VlnPlot. To generate heatmap data of BMEx-overlay populations, mean expression of each gene was calculated per cluster using base R functions, which were then filtered for top 12 differentially expressed genes per cluster (ranking based on highest fold change), and visualized using the pheatmap package (v1.0.12). For the combined analysis with Chen dataset (UCLA2)[11] and with Zheng dataset,[13] we applied the same filtering parameters with regard to global and mitochondrial gene expression as described above. For the combined analysis with the Tyser dataset,[24] we used the filtering parameters used by the authors (number of genes >2,000, percentage mitochondrial <2), and for the combined analysis with the Li dataset,[25] filtering was performed using transcript number between 100,000 and 1,500,000, number of genes >2,000. Batch correction was done on the two replicates in the Chen dataset (UCLA2) using the fastMNN function. For all merged analyses, the same Seurat workflow was applied as for the in-house dataset, with changes in the following parameters: UMAP resolution parameters of the combined analysis with Chen dataset 0.27, with Zheng dataset 0.4, with Tyser dataset 0.3 and with Li dataset 0.4; batch correction was done using the fastMNN function. Heatmap data comparing various germ cell types was generated by calculating mean expression of each gene of interest per cluster of interest and a row-based hierarchical clustering was performed using the ComplexHeatmap package (v2.14.0) with Euclidian distance.

Image quantification and visualization

The quantification of fluorescence intensity was performed using Fiji for image handling and R package ggplot2 (v3.3.5) for data visualization. First maximum intensity projections were generated on Dragonfly confocal image stacks (2048 × 2048 pixels; z-value: 10–12), for three fields of view per analyzed condition. Images were segmented based on DAPI channel to obtain areas representing nuclei. Segmentation consisted of thresholding DAPI signal, generating an image mask, and performing additional segmentation using the Fiji Watershed algorithm to separate overlapping nuclei. ''Analyze particles'' function was used to obtain regions of interest (minimum size set to 40 pixels), and mean fluorescence intensity signals of nuclear markers in other channels were determined in these areas. Data were loaded in R for filtering and visualization using ggplot2 (geom_jitter and geom_violin). Data was filtered for clear outliers that were a result of staining artifacts and autofluorescent debris. In addition, areas above 300 pixels which likely represented multiple nuclei were removed from the analysis.

Cell Reports Methods, Volume *3*

Supplemental information

Efficient and scalable generation of primordial germ cells in 2D culture using basement membrane extract overlay

Arend W. Overeem, Yolanda W. Chang, Ioannis Moustakas, Celine M. Roelse, Sanne Hillenius, Talia Van Der Helm, Valérie F. Van Der Schrier, Manuel A.F.V. Gonçalves, Hailiang Mei, Christian Freund, and Susana M. Chuva de Sousa Lopes

SUPPLEMENTAL INFORMATION

SUPPLEMENTAL FIGURES AND FIGURE LEGENDS

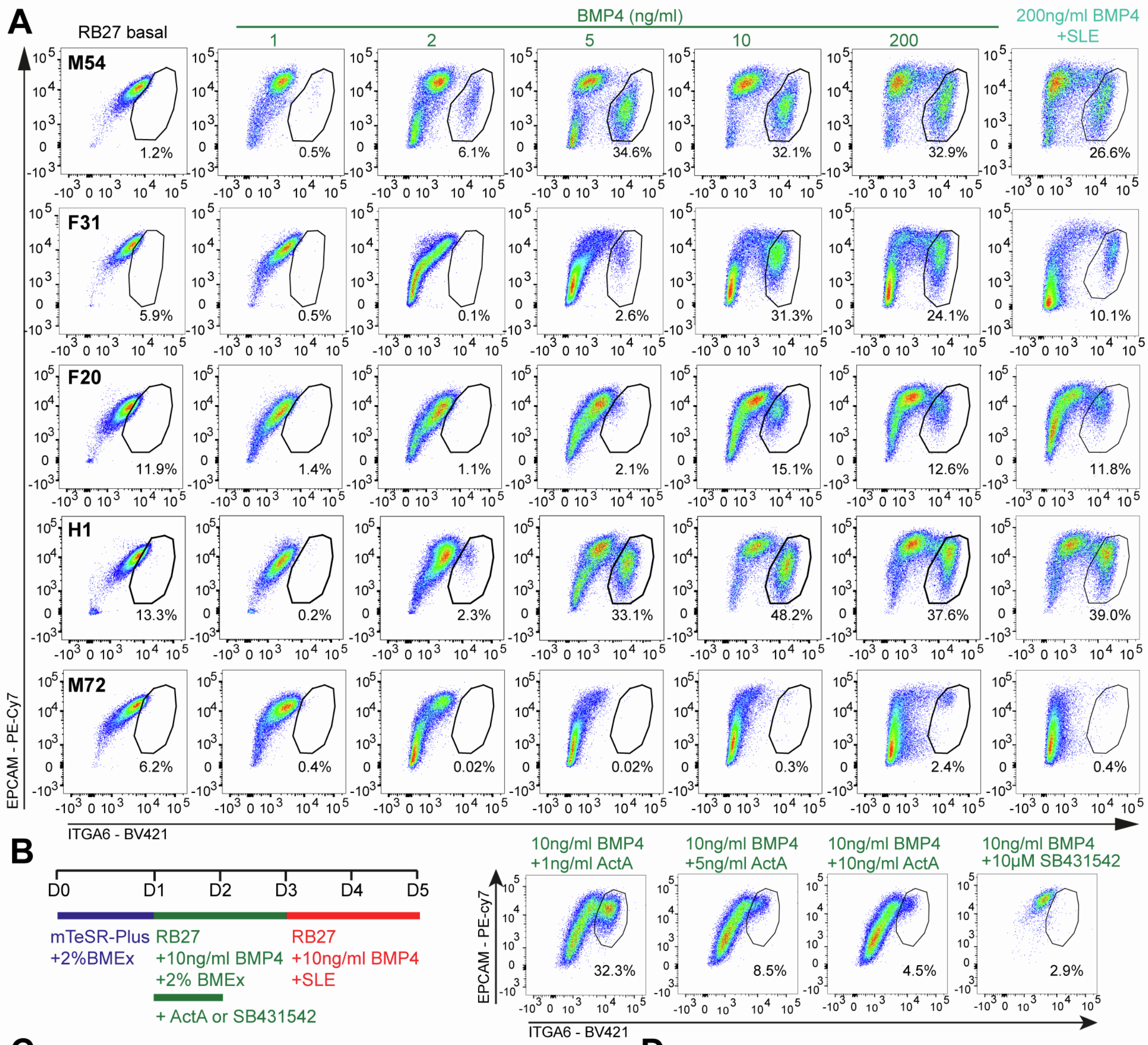

C

	Label	Lot number	BCA assay (mg/ml)	Lowry assay from product sheet (mg/ml)
Geltrex	Lot A	963718	7.0	13.0
	Lot B	963726	5.9	16.0
	Lot C	2327546	6.4	13.2
Cultrex	Lot A	1659223	8.4	9.6
	Lot B	1677279	7.2	9.8
Matrigel	Lot A	1341001	8.8	6.5
	Lot B	1235001	9.1	9.0

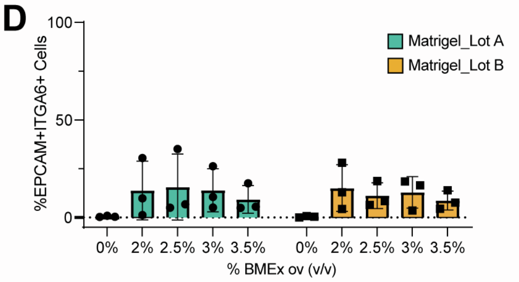

Figure S1. hPGCLC differentiation using BMEx overlay method across multiple cell lines. Related to Figure 2.

(A) FACS plots depicting the percentage of EPCAM+ITGA6+ cells at D5 in different lines to test different BMP4 concentrations.

(B) Experimental scheme depicting different conditions tested (left) and the associated FACS plots (right) depicting the percentage of EPCAM+ITGA6+ cells at D5 in line F99.

(C) Protein concentrations and lot numbers of all the BMEx products used in this study.

(D) Bar graph showing mean percentages (mean ± standard deviation, SD) of EPCAM+ITGA6+ cells at D5 generated using different percentages of hESC-qualified Matrigel overlay comparing to no overlay control (0%), analyzed by FACS in line M54.

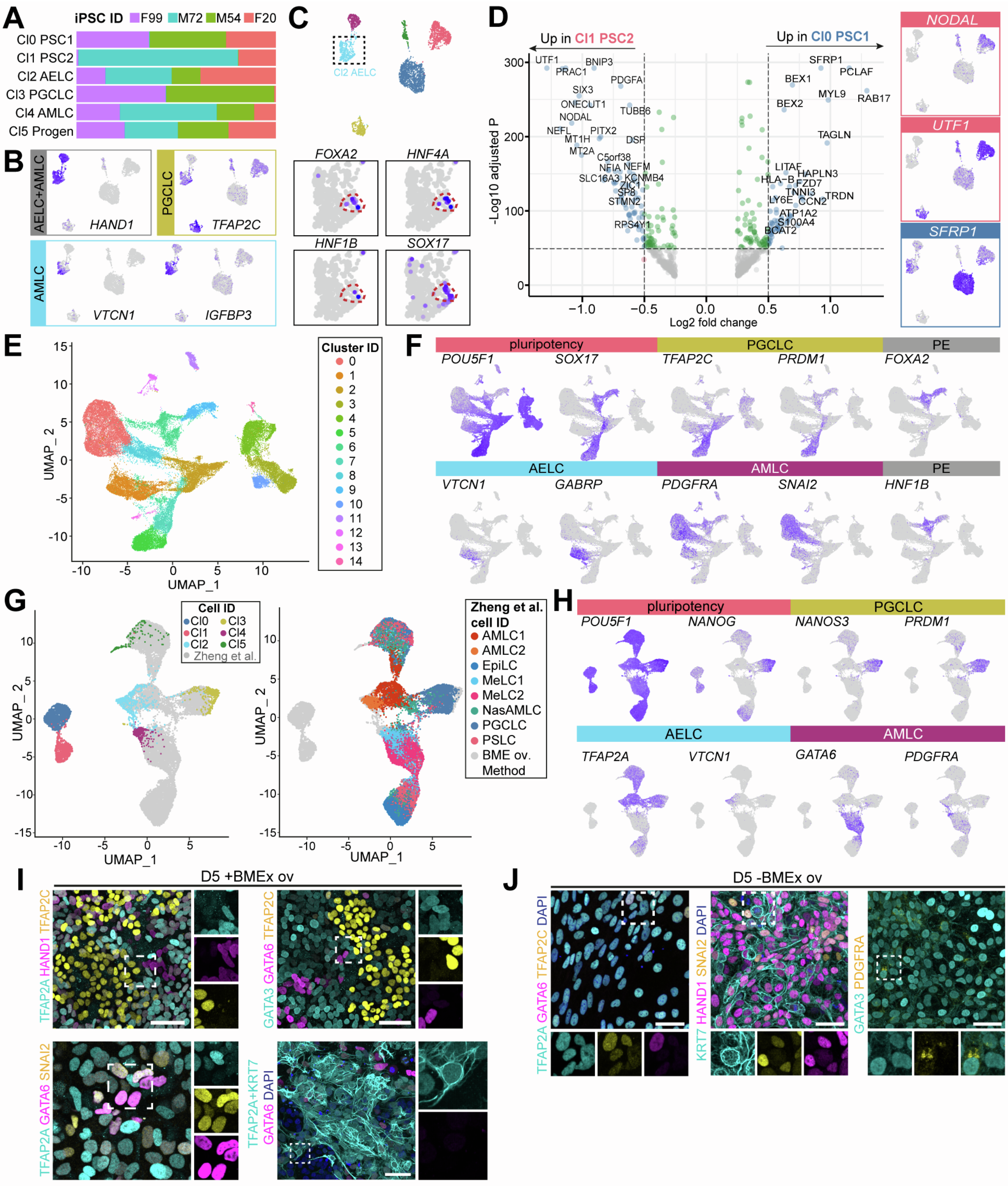

Figure S2. RNAseq comparing BMEx overlay method with EB method and µPASE method, and verification of markers by IF. Related to Figure 3.

(A) Bar graph depicting the contribution per cluster from each cell line.

(B) Expression of additional signature genes of cell types of interest on the UMAP plot from Figure 3A.

(C) Expression of signature genes of endoderm on the UMAP plot from Figure 3A (close up).

(D) Volcano plot showing DEGs between the two D0 hPSCs clusters (Cl0 and Cl1) (left panel) and expression of selected DEGs on the UMAP plot from Figure 3A (right panels).

(E) UMAP showing integrated single-cell transcriptomics data from EB-differentiation method (UCLA2 from Chen et al., 2019) and BMEx overlay method.

(F) Expression of signature genes of cell types of interest on the UMAP plot from Figure 3D.

(G) UMAP showing integrated single-cell transcriptomics data from amniotic sac embryoid system (μPASE) (from Zheng et al., 2022) and BMEx overlay method.
(H) Expression of signature genes of cell types of interest on the UMAP plots from Figure S3G.
(I) Immunofluorescence for TFAP2A, HAND1 and TFAP2C; GATA3, GATA6 and TFAP2C; TFAP2A, GATA6 and SNAI2; and TFAP2A+KRT7 and GATA6 at D5 with BMEx overlay. The dashed box is magnified showing separated channels. Scalebars: 50μm.
(J) Immunofluorescence for TFAP2A, GATA6 and TFAP2C; KRT7, HAND1 and SNAI2; GATA3 and PDGFRA at D5 without BMEx overlay. The dashed box is magnified showing separated channels. Scalebars: 50μm.

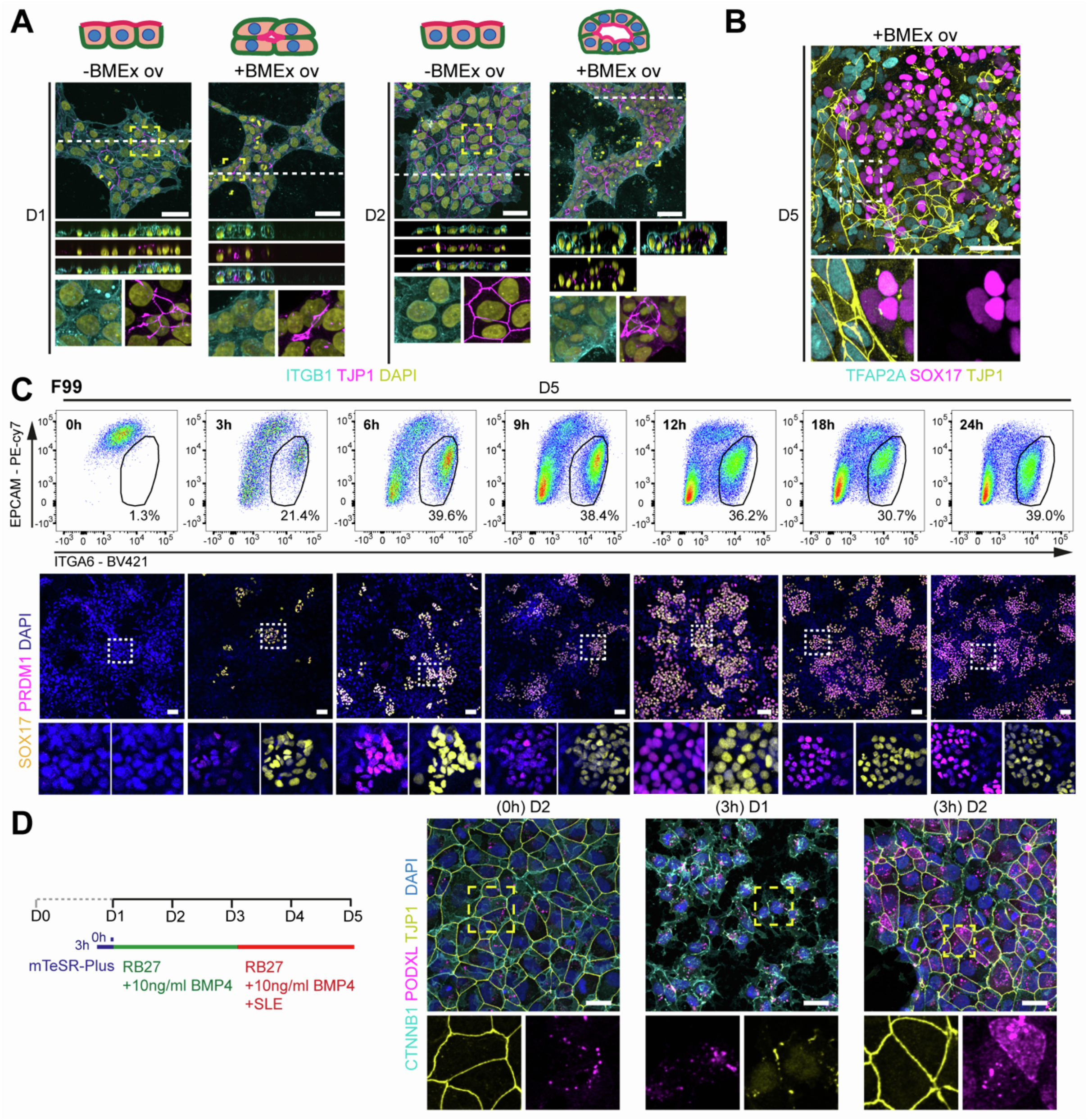

Figure S3. Lumenogenesis during hPGCLC differentiation with BMEx overlay. Related to Figure 5.

(A) Immunofluorescence for ITGB1 and TJP1 at D1 and D2 with or without BMEx overlay. White dashed line shows the level of the digital cross section (middle panels) and yellow dashed box is magnified (bottom) showing separated channels. Scale bars: 30μm.

(B) Immunofluorescence for TFAP2A, SOX17 and TJP1 at D5 with BMEx overlay. Dashed box is magnified (bottom) showing separated channels. Scale bars: 50μm.

(C) FACS plots depicting the percentage of double EPCAM+ITGA6+ cells at D5 in line F99 to test different priming periods (top) and associated immunofluorescence for SOX17 and PRDM1. Dashed box is magnified (bottom) showing separated channels. Scale bars: 50μm.

(D) No BMEx overlay control accompanying Figure 5G. Experimental scheme depicting the conditions tested in the absence of BMEx overlay (left) and immunofluorescence for CTNNB1, PODXL, TJP1 at D1 and D2 (right). Dashed box is magnified (bottom) showing separated channels. Scale bars: 50μm.

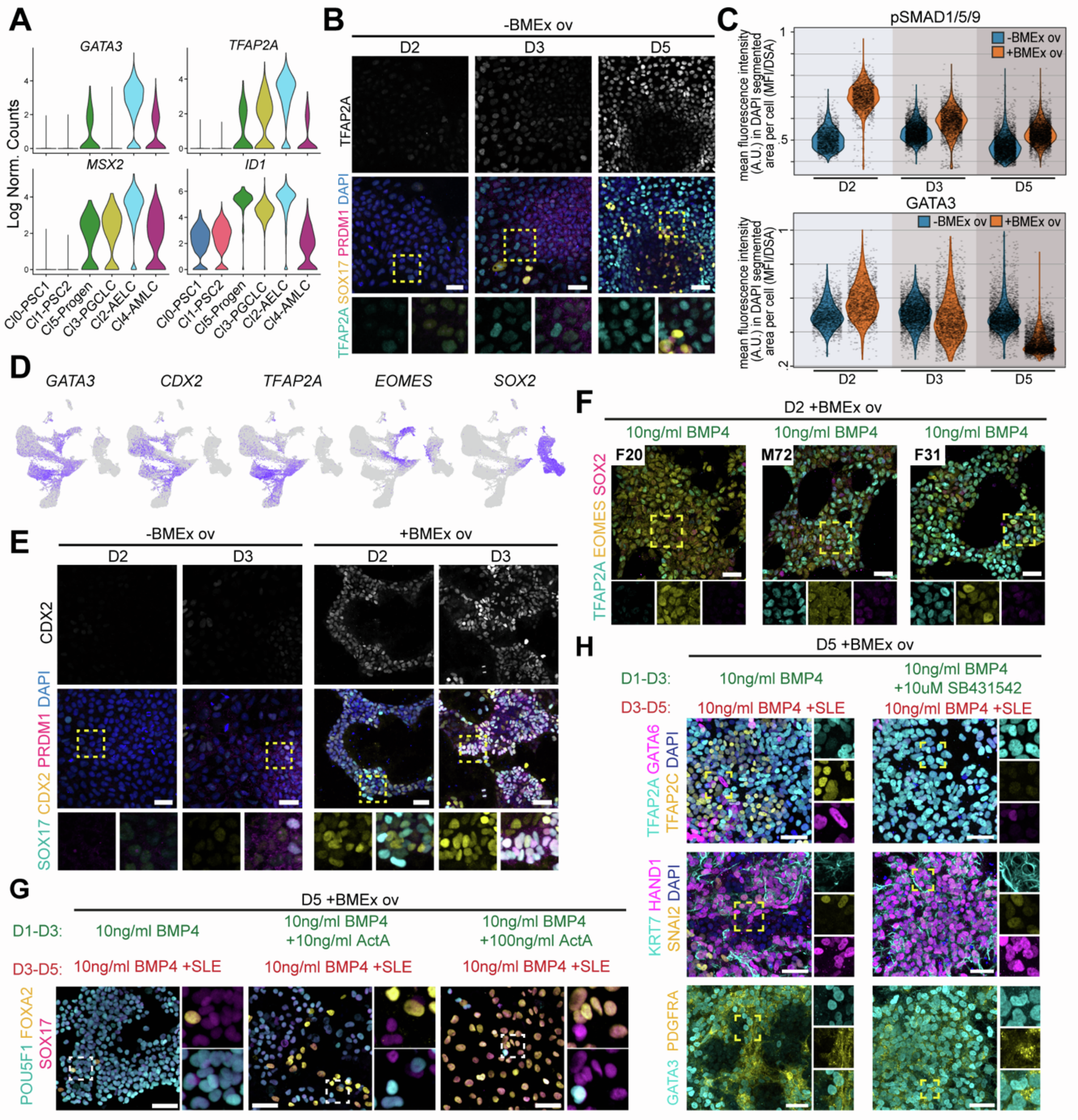

Figure S4. Characterization of D2-progenitors during hPGCLC differentiation with BMEx overlay. Related to Figure 6.

(A) Violin plots showing expression levels of selected genes of interest per cluster.

(B) Immunofluorescence for TFAP2A, SOX17, PRDM1 at D2, D3 and D5 without BMEx overlay in line M54. TFAP2A is shown on top as single channel. Dashed box is magnified (bellow) showing separated channels. Scale bars: 50μm.

(C) Violin plots depict the quantification of the images in Figure 6C as the mean fluorescence intensity in arbitrary units (A.U.) of pSMAD1/5/9 (top) and GATA3 (bottom) in DAPI segmented areas (normalized to 1) per cell.

(D) Expression of signature genes of hPGCLC-progenitors on the UMAP plot from Figure 3D.

(E) Immunofluorescence for SOX17, CDX2, PRDM1 at D2 and D3 with or without BMEx overlay. CDX2 is shown on top as single channel. Dashed box is magnified (bellow) showing separated channels. Scale bars: 50μm.

(F) Immunofluorescence for TFAP2A, EOMES, SOX2 at D2 with BMEx overlay in line F20, M72 and F31. Dashed box is magnified (bellow) showing separated channels. Scale bars: 50μm.

(G) Immunofluorescence for POU5F1, FOXA2, SOX17 at D5 with BMEx overlay in the indicated culture conditions. Dashed box is magnified (right) showing separated channels. Scale bars: 50μm.

(H) Immunofluorescence for TFAP2A, GATA6 and TFAP2C; KRT7, HAND1 and SNAI2; GATA3 and PDGFRA at D5 with BMEx overlay in the indicated culture conditions. Dashed box is magnified (right) showing separated channels. Scale bars: 50μm.
